# Manganese-Doped Carbon Nanodots as Next-Generation MRI and Fluorescence Imaging Agents

**DOI:** 10.1021/acsnano.6c03209

**Published:** 2026-07-20

**Authors:** Michele Cesco, Lydia Martínez-Parra, Samantha Marcelino Apresto, Cecilia Wetzl, Begoña Maria Echeverría-Beistegui, Jesús Ruiz-Cabello, Lucia Cardo, Maurizio Prato

**Affiliations:** † Center for Cooperative Research in Biomaterials (CIC biomaGUNE), Basque Research and Technology Alliance (BRTA), Paseo de Miramón 194, Donostia-San Sebastián 20014, Spain; ‡ University of the Basque Country UPV-EHU, Donostia-San Sebastián 20018, Spain; § Ikerbasque, Basque Foundation for Science, Bilbao 48013, Spain; ∥ Centro de investigación en red de enfermedades respiratorias (CIBERES), Instituto de Salud Carlos III, Madrid 28029, Spain; ⊥ Departamento de Química en Ciencias Farmacéuticas, Universidad Complutense de Madrid, Madrid 28040, Spain; # Dipartimento di Scienze Chimiche e Farmaceutiche, 9315 Università degli Studi di Trieste, Trieste 34127, Italy

**Keywords:** carbon nanodots (CNDs), manganese-doped nanoparticles, T1-weighted MRI, nanomedicine, nanoprobes for biomedical imaging, manganese contrast agents

## Abstract

Carbon nanodots (CNDs) have emerged as highly promising nanomaterials for biomedical applications due to their intrinsic fluorescence, excellent biocompatibility, and facile surface functionalization. Doping CNDs with paramagnetic metals, such as manganese (Mn), enables the development of dual-modal imaging probes that integrate magnetic resonance imaging (MRI) and fluorescence imaging (FI). Mn is a particularly attractive alternative to Gd-based contrast agents, offering lower toxicity, favorable paramagnetic properties, and physiological relevance. Here, we report a rapid microwave-assisted hydrothermal synthesis of Mn-doped carbon nanodots (MnCNDs) as dual-mode contrast agents for MRI and FI. A size exclusion chromatography purification step yielded a uniform population of MnCNDs containing 5% (w/w) Mn­(II), characterized by excitation-dependent fluorescence emission and an amorphous carbon structure with a metal-enriched core. Importantly, MnCNDs exhibit a stable longitudinal relaxivity over 7 days, comparable to that of clinically used MRI contrast agents. Confocal fluorescence imaging confirmed efficient cellular uptake in vitro, while T1-weighted MRI in mice demonstrated their biocompatibility and strong potential as effective MRI contrast agents for *in vivo* applications. These findings position MnCNDs as robust and versatile nanoprobes for next-generation, multimodal bioimaging.

## Graphical abstract

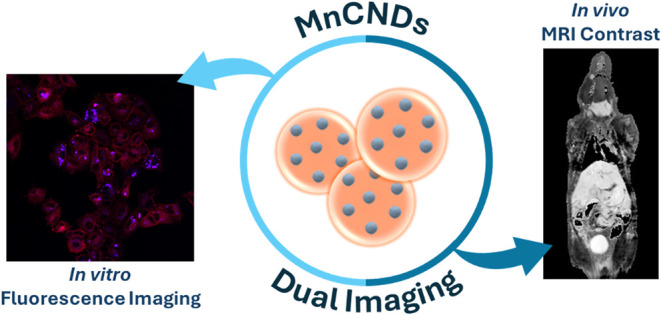

Contrast agents (CAs) are pharmaceuticals routinely employed in clinics to enhance the visibility and diagnostic accuracy of various medical images.^1^ By altering the contrast between different tissues or structures, CAs help delineate anatomical details and identify pathological changes more effectively. In magnetic resonance imaging (MRI), protons, primarily from water molecules in the body, are excited by radiofrequency (RF) pulses in the presence of a strong magnetic field. The resulting relaxation of the proton spins to the initial state generates RF signals that are detected and translated to images. This relaxation process can occur in two modes, through (i) longitudinal relaxation (recovery along the magnetic field axis), characterized by relaxation time T_1_, and (ii) transversal relaxation (loss of phase coherence in the perpendicular plane), characterized by relaxation time T_2_. Both T_1_ and T_2_ are affected by different local microenvironments in biological systems; therefore, they vary across different tissue types, and this is employed to generate several contrasts in MR images.^2,3^ However, intrinsic contrast is not always satisfactory, and for some tissue or occurring pathology, relevant medical information may be lost. In these cases, CAs for MRI are employed: they work as magnetically active compounds that alter the magnetic environment of specific biological targets or events, thereby affecting T_1_ and/or T_2_ and enhancing contrast between tissues or highlighting the presence of pathologies.^4^

Currently, CAs employed for MRI are gadolinium III (Gd (III)) metal complexes such as Gd-DTPA (Magnevist), Gd-DOTA, (Dotarem), Gd-DO3A-butrol (Gadovist), Gadodiamide (Omniscan) and few others.^5,6^ The dominant role of paramagnetic Gd (III)-based CAs (GbCAs) is related to the electronic configuration of this rare earth metal (seven unpaired electrons when in oxidation state +3), strongly affecting (shortening) T_1_ of protons from coordinating water molecules. As a result, tissues probed with GbCAs are brighter (positive contrasts). In addition, these complexes, based on multidentate ligands, are rather stable and, below certain dosages, they have been considered suitable for this application for decades. Nevertheless, safety issues have emerged regarding the accumulation of Gd^3+^ in organs, such as brain or kidneys,^7,8^ especially in patients with impaired renal function. As an additional concern, the environmental persistence of GbCAs has been recently documented.^9^ Overall, the demand for safer and more sustainable CAs is growing, and, at the same time, there is a technological request for a next-generation of imaging tools, not only for improved safety profiles but also for multifunctionality, including theranostic capabilities and multimodal imaging compatibility (e.g., MRI/optical or MRI/PET). Among the many alternative CAs explored over the past decades, superparamagnetic iron oxide nanoparticles (SPIONs)^10^ and manganese-based compounds^11,12^ have been the closest to clinical translation. This is due to their favorable biocompatibility, competitive contrast efficiency, and suitable biodistribution, making them viable alternatives to gadolinium agents in both diagnostic and theranostic applications. Focusing on paramagnetic manganese-based CAs (MBCAs), the metal in oxidation state +2 holds five unpaired electrons and a coordination number of five, six, or seven. The most mature Mn compounds alternatives to Gd agents are small Mn (II) complexes based on penta- or hexa-dentate ligands, leaving at least one site in the inner coordination sphere available for water molecules exchanging at a fast rate. The defined and controlled molecular structure of these mononuclear Mn (II) complexes, their competitive relaxivity properties, and favorable clearance are an advantage; at the same time, their rapid nonspecific pharmacokinetics, modest multifunctionality, lower thermodynamic stability, and kinetic inertness compared to GbCAs, have limited their acceptance in clinics so far.^13^ In this context, nanotechnology is a valid and widely explored alternative approach. In particular, nanoparticles (NPs) of different nature (both organic and inorganic) or biocompatible polymeric systems have been doped with Mn (II) to achieve multimodal nanosystems with significantly improved magnetic properties and enhanced stability and are able to bear other functions such as therapeutic activity (chemical or light responsive) or additional imaging modes. Mn oxide and related inorganic nanosystems have shown high Mn payload with enhanced T_1_, surface tunability, and multifunctionality, although their broader translation is still limited by structural heterogeneity, reticuloendothelial uptake, and the need to better control Mn release and long-term safety^11,14,15^

In this work, carbon-based nanoparticles, specifically carbon nanodots (CNDs),^16^ were employed as a platform for the development of Mn­(II)-loaded systems that are stable, safe *in vivo*, and capable of acting as T_1_-weighted MRI contrast agents. CNDs are quasi-spherical nanoparticles with typical diameters in the range of 3–10 nm, and due to their carbon-based backbone, they are classified as low-dimensional components within the broader family of carbon-based nanomaterials. Owing to their remarkable structural and chemical versatility, CNDs have emerged as promising materials for a wide range of biorelated applications, including bioimaging, sensing, drug delivery, theranostics, and light-triggered therapies.^17–20^ They are typically water-soluble by design, without requiring postsynthetic surface modification, and exhibit good stability in biologically relevant environments together with favorable *in vivo* safety profiles. Furthermore, rational synthetic approaches enable the incorporation of accessible surface functional groups, facilitating postsynthetic bioconjugation strategies.

CNDs are commonly obtained through bottom-up synthetic routes, in which selected organic precursors undergo carbonization under solvothermal conditions to yield nanoscale particle-like frameworks.^21^ Within this approach, metal ions can be introduced into the precursor mixture,^22,23^ leading to metal-doped CNDs with typically low metal loadings (≈3–15%) stabilized within the particle framework. Overall, successful formulations of Mn-doped CNDs are attractive candidates to address current limitations in the state-of-the-art. In particular, the stable incorporation of Mn within a robust carbon framework may represent a significant advancement over the limited thermodynamic stability and kinetic inertness of conventional Mn-complexes. At the same time, MnCNDs combine the high surface area, tunable physicochemical properties, and imaging performance typical of nanomaterials with an ultrasmall carbon-based structure that may mitigate some drawbacks of larger nanoparticles, including poor biodegradability, long-term accumulation, and limited clearance.

Several studies have reported CNDs doped with paramagnetic ions such as Mn, Gd, or Fe as MRI contrast agents, with observable *in vivo* signal enhancement.^18,24–26^ Although these reports highlight the potential of metal-doped CNDs for biomedical imaging, important challenges remain that limit their translational relevance. In particular, concerns have increasingly emerged regarding sample purification and analytical reliability.^27–29^ Standard purification protocols relying solely on filtration and dialysis may be insufficient, as small fluorophores, molecular byproducts, or low-molecular-weight metal complexes generated during synthesis can persist and substantially contribute to the observed optical or magnetic resonance signals. This complicates the attribution of MRI contrast, affects reproducibility, and raises questions regarding toxicity assessment and biological interpretation.

Here, we established a new Mn­(II)-doped CND platform and identified new synthetic, analytical, and purification criteria to improve yield, reliability, and sample quality. We selected high-performance liquid chromatography (HPLC) combined with size exclusion chromatography (SEC) as a method to isolate 5–8 nm Mn-doped nanoparticles from fluorescent artifacts with different size distributions and charge as well as Mn ions or Mn adducts that were not stably included in the particle framework. This purification approach, novel for Mn-doped CDs (to the best of our knowledge), ensured (i) the elimination of fluorescent large artifacts able to resist dialysis and influencing the optical and relaxiometric properties of the material; (ii) the elimination of unwanted Mn-based species and improved homogeneity and quality of the final formulation.

Beyond materials characterization, we specifically addressed the *in vitro* and *in vivo* behaviors of MnCNDs under physiologically relevant conditions. To date, only a limited number of studies have investigated MnCNDs *in vivo* as MRI contrast materials, with most focusing on cancer imaging and therapy.^30–35^ In most cases, signal detection relies on intratumoral injection,^34,35^ which does not reflect realistic biodistribution or systemic pharmacokinetics. Two recent relevant studies have reported *in vivo* imaging studies upon intravenous administration, both within a therapeutic context: in one interesting case, MRI contrast was enhanced in the targeting organ following photoinduction,^30^ while in the other, contrast enhancement was associated with specific tumor targeting,^28^ but it was not employed during standard biodistribution studies. In contrast, the Mn-CND system developed here is clearly detectable by MRI following intravenous administration (1–2 h postinjection) during standard biodistribution, without requiring tumor accumulation, active targeting, or external activation. This distinguishes our study from previous reports and demonstrates the potential of MnCNDs as versatile and general-purpose MRI contrast agents. While the system remains potentially compatible with future biotargeting or light-induced therapeutic strategies, the present work intentionally focuses on indicating new synthetic and purification criteria to improve formulation reliability, followed by *in vitro* and *in vivo* characterization. This last included fundamental biodistribution, short and long-term toxicity, and intrinsic contrast performance, providing a robust reference framework for future translational development.

## Results and Discussion

### Synthesis and Purification

We recently reported that fluorescent CNDs doped with Gd (III) could function as T_1_ contrast agents *in vivo*.^36^ The nanoparticles showed high stability, appropriate excretion pathways and timing, and good imaging efficiency, without the need for specific biotargeting, which is often required to enhance the material accumulation and signal strength of doped NPs. Gd-CNDs were obtained using β-Alanine, ethylenediaminetetraacetic acid (EDTA), and GdCl_3_ as precursors for the microwave-assisted solvothermal reaction. This was our starting reference for designing analogous CNDs doped with Mn, but we also explored the possibility of improving the synthetic protocol.

In our former design, β-alanine served as a zwitterionic source of nitrogen, useful for achieving enhanced emission intensity in the blue region, while EDTA worked as a polyreactive linker, useful for assembling the carbonaceous platform and chelating the metal. Keeping these two precursors in mind, we employed manganese chloride tetrahydrate (MnCl_2_·4H_2_O) as the metal source and introduced glutaraldehyde (50% solution in water) as the third organic precursor to verify whether its reactivity toward nucleophilic groups in the mixture would favor the cross-linking (Figure 1). Two reactions, with and without glutaraldehyde, were carried out in a microwave at 240 °C under fixed power conditions (75 W, 300 PSI pressure), followed by filtration, dialysis, and freeze-drying, to obtain two crude powders (cMnCNDs). We observed that both compounds contained comparable amounts of Mn (8–9% w/w measured by ICP-MS analysis), but when glutaraldehyde was used, the yield of the reaction increased (from 9.3 to 15.2% mass yield). This implies that the third reagent in the mixture did not significantly affect the loading of the metal, but it boosted the carbonization process related to the generation of the carbon framework, and the higher yield achieved (∼2-fold) was a relevant improvement in the synthetic procedure (see also the Supporting Information, SI). We and others have reported that additional purifications (beyond dialysis) are essential for many CND formulations, to guarantee the reliability of the material and avoid misinterpretations concerning their activity and performance.^28,36–38^ Also in this case, gel electrophoresis (GE) analysis of cMnCNDs (after dialysis) revealed a rather heterogeneous composition of the crude compound, as a mix of both positively and negatively charged populations (Figure S1E). ICP-MS analysis showed that only the negative population was loaded with Mn, while the positive impurities were emissive artifacts, and their removal was required. For purification, we employed size exclusion chromatography coupled with high-performance liquid chromatography (SEC-HPLC), using NH_4_^+^HCO_3_^–^ buffer (pH 8.5) as the eluent.^39^ Fluorescence and absorbance chromatograms and the criteria for the collected fractions are shown in Figure S1C,D. Shortly, the fraction collected included only the negatively charged and Mn-doped fluorescent population, selected as the purified material and named pMnCNDs (see GE analysis in Figure S1E and z-potential characterization in Figure S1G). Presumably, the material with lower molecular weight, eluted at higher retention times, corresponded to positively charged fluorescent impurities and was therefore excluded from the final formulation. The purified pMnCNDs were further characterized, and some analyses were performed to highlight the relevance of the purification process by comparison with cMnCNDs.

**Figure 1:**
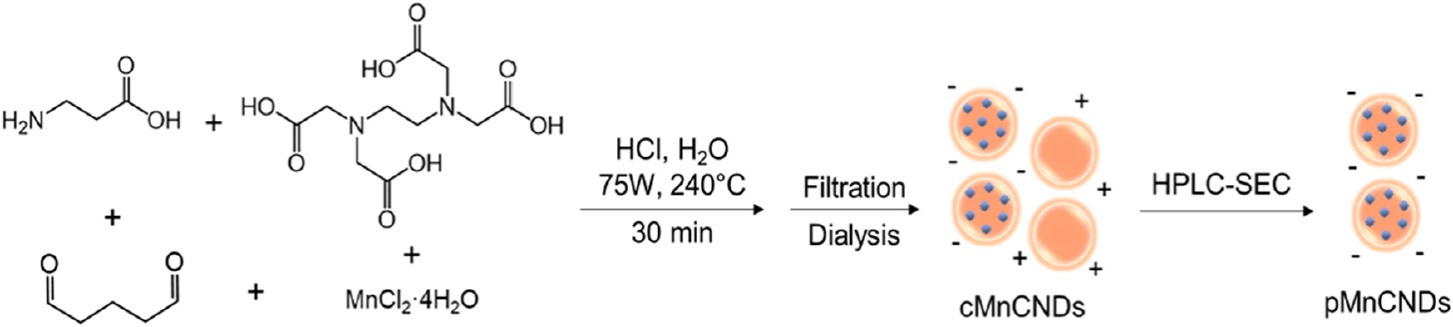
Synthesis of manganese-doped CNDs (MnCNDs) using a microwave-assisted method. The crude product (cMnCNDs) was obtained by filtration, dialysis, and freeze-drying. The final material (pMnCNDs) was obtained by purification using SEC-HPLC (see Figure S1).

### Physicochemical Characterization of pMnCNDs

pMnCNDs contained 5% w/w Mn, as measured by ICP-MS. The absorption spectra of both the cMnCNDs and pMnCNDs were primarily localized in the UV region (Figures 2A and S2A, respectively) with very similar profiles (see also Figure S1F for a macroscopic comparison of the material before and after purification). CNDs are well-known for their intrinsic excitation-dependent emission properties, commonly attributed to the heterogeneous distribution of emissive states within the carbon framework, including core-associated domains and chemically diverse surface states.^40,41^ This strictly depends on the selection of precursors, solvents, and reaction conditions employed. As expected, both cMnCNDs and pMnCNDs exhibited emission predominantly in the blue visible region (Figures 2B and S2B, respectively), although differences between the two compounds were observed.

**Figure 2:**
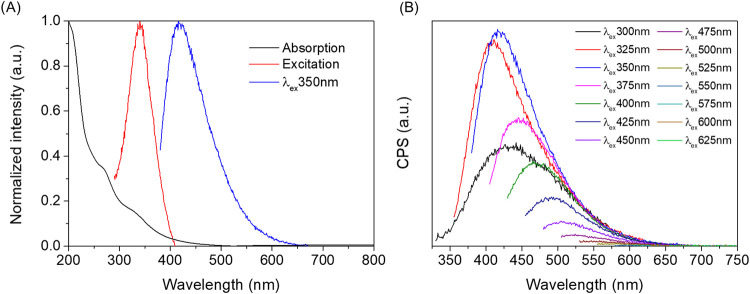
(A) Absorption (black), excitation (red), and emission (λ_ex_ = 350 nm) of the pMnCNDs. (B) Fluorescence map of pMnCNDs.

The excitation spectrum of the crude product showed a maximum at 380 nm, whereas that of the purified product shifted to 340 nm. Consequently, the emission profiles also differed: cMnCNDs displayed maximum emission intensities in the 450–525 nm region (upon excitation in the 380–425 nm range), whereas the emissions of pMnCNDs with higher intensities were blue-shifted within the 400–425 nm range upon excitation at 325–350 nm. The quantum yields (QY) of cMnCNDs and pMnCNDs, measured at 350 nm excitation, were 7 and 10%, respectively. All this confirms the importance of the applied purification process, which enables the removal of fluorescent Mn-free side products,^27,42^ yielding more homogeneous and reliable emission profiles. Atomic force microscopy (AFM) and transmission electron microscopy (TEM) were performed to determine the size and morphology of pMnCNDs. AFM images of both cMnCNDs and pMnCNDs showed the presence of spherical materials; however, in the case of the crude compound, the size distribution obtained upon particle analysis was wider (Figure S3A,B), with a higher percentage of low-sized material. In comparison, the size distribution of pMnCNDs was reduced by an average of 7 nm (Figure 3A,B, respectively), confirming that smaller components were eliminated and size homogeneity improved upon purification. TEM images of pMnCNDs also revealed the presence of quasi-spherical particles, although an average size of approximately 4 nm was measured in this case (Figure 3C,D). The different size distributions detected by AFM and TEM could be either an actual difference between the z dimension (measured by AFM) and the *x*/*y* plane observed in TEM analysis, or just related to the different contrasts of the material with these two techniques. This latter may be the case for low-dimensional particles with an amorphous carbon framework, whose contrast in TEM images is expected to be poor. High-resolution transmission electron microscopy (HR-TEM) was performed (Figure 3E). Particles were clearly observed, and in some localized regions, lattice fringes could be identified. However, these ordered domains were not uniformly distributed, and the overall structural appearance suggests that the material is predominantly amorphous. This is consistent with the XRD data discussed below. It should also be considered that electron beam exposure during HR-TEM analysis may induce local structural rearrangements in carbon-based materials.^43^ The beam does not solely image the sample but also deposits energy, which can promote partial ordering or graphitization, particularly in small and initially poorly ordered carbon systems. Indeed, a control experiment showed that during HR-TEM acquisition, prolonged beam exposure caused changes in sample morphology, and increased graphitization was observed (Figure S4A). Therefore, the presence of limited lattice fringes should be interpreted with caution and does not necessarily indicate a fully crystalline structure. Additional images of the material were obtained by using high-angle annular dark-field scanning transmission electron microscopy (HAADF-STEM, Figures 3F and S4), and the generated particle size distribution was comparable to that obtained by AFM (average size of ∼7 nm, Figure 3G), supporting the hypothesis that the contrast observed in TEM might primarily arise from the Mn-dense area, while the entire dimensions of these carbon particles are better identified by AFM and STEM techniques. Interestingly, in a significant fraction of the particles examined in HAAD-STEM images, brighter regions surrounded by areas of lower intensity were observed. Given that HAADF contrast approximately scales with atomic number (Z-contrast), this intensity variation is consistent with regions of relatively higher average atomic number, potentially associated with Mn-enriched domains, embedded within a carbonaceous framework. However, contrast alone cannot unambiguously confirm elemental identity, particularly in ultrasmall (4–7 nm), amorphous carbon-based systems. To further support this interpretation, we performed EDX line-scan analysis, which was feasible only across selected particle clusters, since the reduced size and the high beam sensitivity of the particles during scan acquisition did not allow single particle analysis (Figure S4C). Nevertheless, Mn was detected in EDX, and the line-scan analysis showed colocalization of Mn with C and N, with the Mn signal spatially confined within the carbon-based framework and extending over a smaller region compared to the carbon signal. These results support the association of Mn with the organic framework.

**Figure 3:**
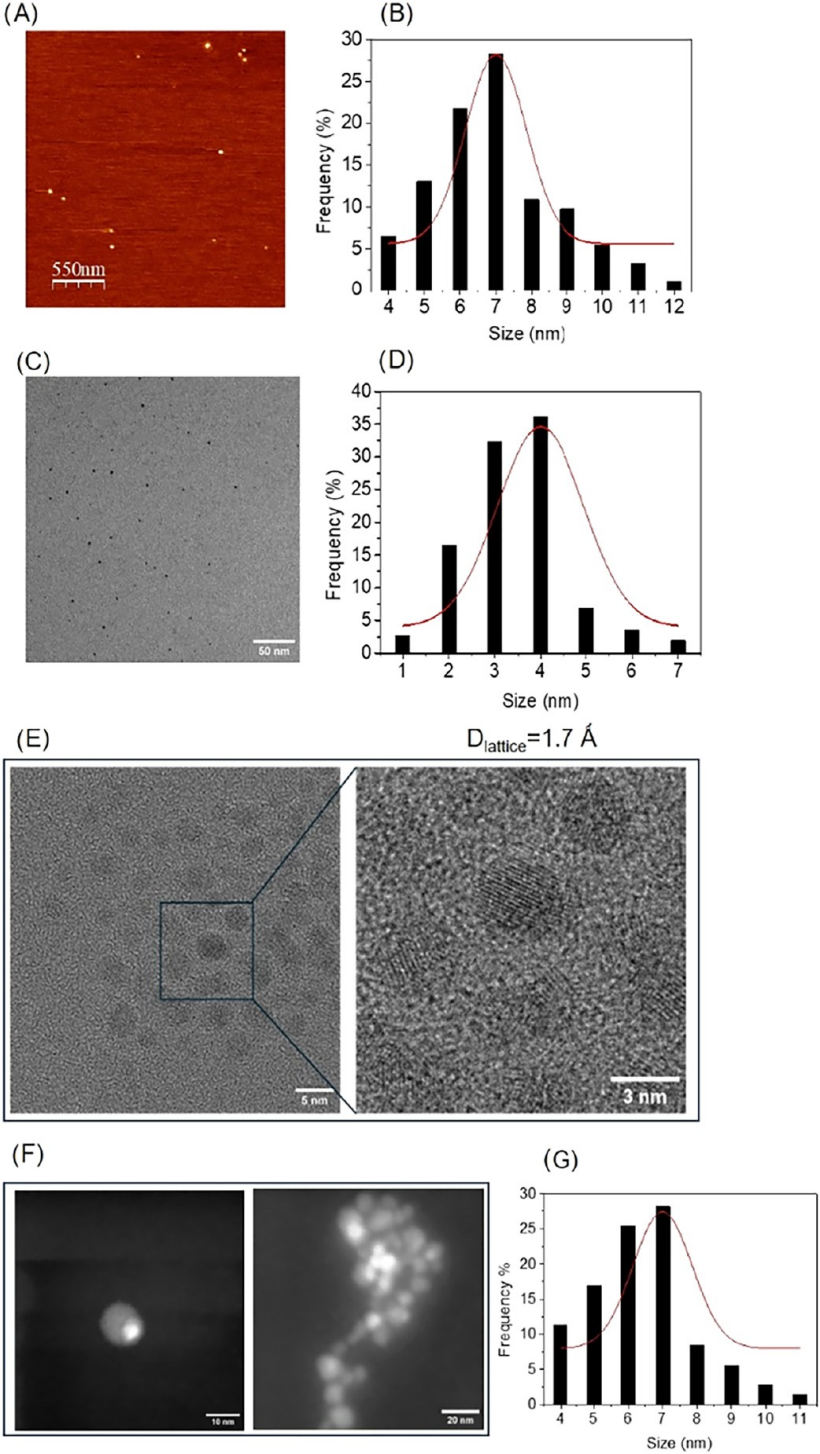
pMnCND morphology characterization. (A) AFM images and (B) related average size distribution; (C) TEM images and (D) average size distribution. (E) HR-TEM images with enlarged representative region; (F) 2 representative STEM images showing areas with brighter contrast, possibly corresponding to metallic core surrounded by an organic framework (weaker contrast areas), and (G)-related size distribution (additional EM images in Figure S4).

^1^H NMR analysis is usually employed to detect the presence of side products in CDs mixtures,^28^ but this is not very informative in this case, as the spectra were highly disturbed by the presence of the paramagnetic metal. Both cMnCNDs and pMnCNDs show very similar profiles (Figure S5A), with broad signals centered at 3.9 and 4.2 ppm, along with a broad band ranging from 3.8 to 1 ppm, while peaks possibly ascribed to molecular impurities were not detected. The composition of pMnCNDs was investigated using X-ray photoelectron spectroscopy (XPS). The survey spectrum (Figure 4A) shows the presence of C (68.0%), N (16.2%), O (7.8%), and Mn (7.9%), confirming the purity of the material. By converting the atomic to mass percentage, the technique revealed an Mn w/w of 4.7%, corroborating the values obtained from the ICP-MS analysis. The metal environment was analyzed by examining the Mn_3s_ and Mn_2p_ regions (Figure 4B,C, respectively). The first Mn_3s_ region enables the straightforward identification of the metal oxidation state^44^ by observing the binding energy difference between the two peaks. Magnitude of peak splitting ΔEMn_3s_, at 82.8 and 88.8 eV, respectively, resulted to be 6 eV, suggesting the presence of Mn (II) species. Mn_2p_ orbital generates two peaks at 641.5 and 652.7 eV and a satellite feature at 645.5 eV. The latter confirms the oxidation state Mn­(II).^44,45^ Carbon 1s signal (Figure S6A) at 285.0 eV includes four components with the following binding energies and bond designations: 284.8 eV (C–C/C–H bonds), 285.9 eV (C–O bond), 287.6 (O–C–O bonds), and 288.5 (O–C=O bonds) eV, the last three being consistent with the presence of carboxylic groups. Oxygen 1s deconvolution displays two components at 531.2 and 533 eV related to C–O and C=O bonds, respectively. (Figure S6B). Finally, the nitrogen 1s spectrum shows two components at 399.7 and 400.6 eV, attributed to the pyridinic C=N bond and pyrrole C–N bond (Figure S6C). As a comparison, we collected the XPS spectrum of cMnCND (Figure S6D–G). An analysis of the Mn_3s_ and Mn_2p_ regions showed no relevant differences relative to the pure compound, while a comparison of the C 1s regions suggests that differences in the carbon-related framework occur (i.e., the component associated with C–C/C–H bonds displays a stronger contribution). Although XPS fitting and peak deconvolution inherently involve a degree of arbitrariness, these observations are consistent with the results obtained from optical spectroscopy, AFM, FT-IR, and ICP-MS analyses. Altogether, the data suggest that the side products are likely positively charged, particle-like materials whose carbon matrix primarily influences the optical properties, total charge, and the relaxivity behavior (see the Relaxivity and Stability section below).

**Figure 4:**
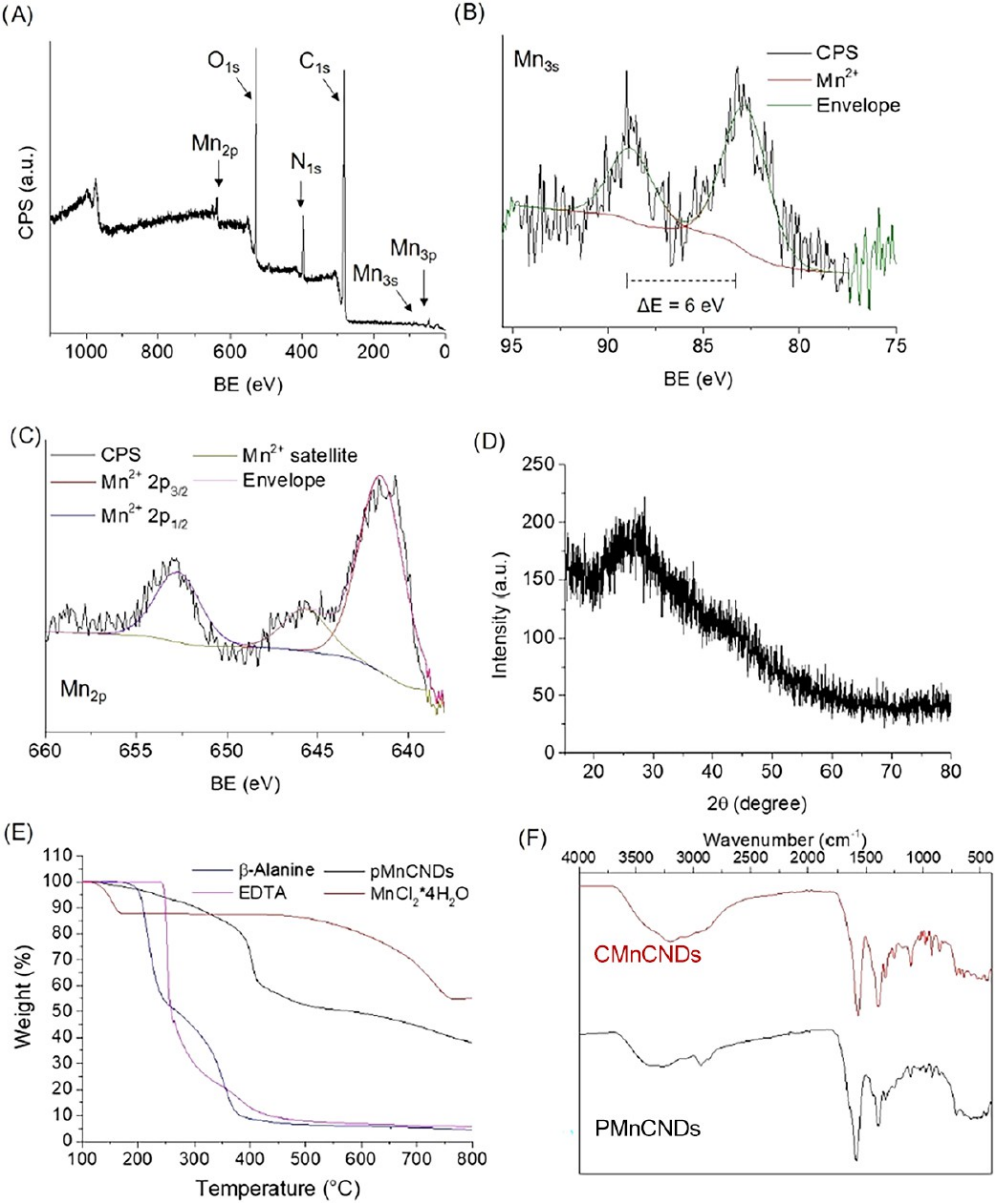
(A) XPS survey spectrum of pMnCNDs showing all of the elements present during the analysis. (B) and (C) Deconvolution and curve fitting of Mn_3s_ and Mn_2p_. (D) XRD spectrum of pMnCNDs. (E) TGA thermograms of pMnCNDs compared to the precursors under an inert atmosphere. (F) FT-IR spectra of cMnCNDs and pMnCNDs.

X-ray diffraction spectroscopy (XRD) (Figure 4D) of pMnCNDs was performed, showing a pattern with two wide bands rather than discrete Bragg reflections, indicating a predominantly disordered carbon structure, consistent with TEM observations. The main band at 2θ ≈ 27° is attributed to the (002) stacking of graphitic layers with enlarged interlayer spacing and limited coherence length, typical of nanosized sp^2^ domains.^46,47^ The broad feature centered at 2θ ≈ 42° is also in agreement with previous reports on amorphous carbon dots.^39,48^ No sharp reflections attributable to crystalline inorganic phases are observed, excluding secondary crystalline components; therefore, this signal is interpreted as a diffuse scattering band rather than a true Bragg reflection. This is commonly associated with in-plane (100)/(101) correlations of turbostratic graphitic carbon, reflecting short-range ordering within small sp^2^ aromatic domains. The absence of higher-order graphitic reflections further supports a turbostratic/amorphous carbon framework.

TGA was performed to establish the thermostability of pMnCNDs (Figure 4E). Starting materials, EDTA and β-Ala, degrade completely through rapid weight drop stages (typical of small organic molecules). MnCl_2_·4H_2_O shows a first weight loss up to 160 °C, associated with MnOHCl generation, which is finally converted to manganese oxides species.^49^ pMnCNDs shows a gradual degradation of 20% of the weight, followed by a little weight drop up to 60%, and another gradual weight loss. The profile suggests the presence of about 40% of oxygen and nitrogen-containing groups, which are degraded before reaching 500 °C.^50,51^ The remaining fraction, consisting of carbon and manganese, is gradually reduced to 40% of its initial weight at 800 °C. Glutaraldehyde’s boiling point is 187 °C, making running TGA measurements impossible. The FT-IR spectra of cMnCNDs and pMnCNDs were comparable (Figure 4F), both showing broad bands at 3675–2340 cm^–1^ resulting from the stretching vibration of C–H, N–H, and O–H bonds. Additionally, pMnCNDs exhibits a minor signal at approximately 2930 cm^–1^, probably related to aliphatic stretching of CH_2_ and CH_3_ groups. The band around 1570 cm^–1^ is ascribed to the stretching vibration of C=O of O=CO– groups, indicating that both the materials possess deprotonated carboxylic acid, probably involved in metal chelation. A weak signal is observed at 1440 cm^–1^, which can be produced by the aliphatic vibration of CH_2_ and CH_3_ groups. The absorption band at about 1390 cm^–1^ arises from the stretching modes of C–O, C–C, and C–N of the amorphous organic framework. The band at around 1230 cm^–1^ belongs to the stretching vibration of C–N, while the peak at 1055 cm^–1^ is related to C–O stretching vibrations. Finally, a standard acid–base titration was performed, confirming the acidic nature of pMnCNDs, with a p*K*_a_ of 3.3 (Figure S7B).

### Relaxivity and Stability

To establish their suitability as CAs, the relaxometric properties of pMnCNDs were evaluated. Longitudinal (r_1_) and transversal (r_2_) relaxation rates were measured at different concentrations using two magnetic fields at 1.5 and 7 T (Figures 5A and S8A, respectively). At 1.5 T, pMnCNDs exhibit an r_2_/r_1_ ratio below 3, confirming their potential as T_1_-weighted CAs. However, at 7 T, the r_2_/r_1_ ratio increased to 4.5, suggesting a possible dual T_1_/T_2_ imaging capability with a stronger effect on T_1_ relaxation. Indeed, effective T_2_ CAs typically display r_2_/r_1_ ratios above 5 (Figure 5B). It is worth mentioning that the measurements at 1.5 and 7 T were performed at different temperatures (37 °C and room temperature, typically 22 °C, respectively), due to different instrumental setup (see the method in the SI). As temperature is known to impact the relaxation process by altering molecular motion and interaction dynamics, this likely explains the variation in r_2_/r_1_ ratios observed between the two magnetic fields.^52,53^ At the same time, the increase of the r_2_/r_1_ ratio at higher fields is expected for most paramagnetic T_1_ contrast agents, including Mn- and Gd-based systems, and mainly reflects the field dependence of relaxation mechanisms.^54^ In any case, the resulting longitudinal relaxivity (r_1_) is 6.24 mM^–1^ s^–1^ at 1.5 T, which falls within the range reported for clinically used GbCAs. For comparison, the r_1_ value of commercial Gadovist, measured here as a control under the same conditions, was 3.54 mM^–1^ s^–1^. More broadly, considering the relaxivities of clinically used GbCAs (extensively reported in the literature^55^), the magnetic properties of pMnCNDs are consistent with potentially detectable MRI contrast. This is an encouraging although preliminary indication, since *in vivo* performance is determined by additional factors beyond relaxivity alone (i.e., biodistribution, stability, metal accessibility, tissue accumulation, etc.). Interestingly, the r_1_ of pMnCND increased after purification, becoming approximately 2-fold higher than the relaxivity of cMnCND (r_1_ = 2.99 mM^–1^ s^–1^, Figure S8H–K), and it was also ∼2.3-fold higher than the r_1_ of free Mn^2+^ ions (r_1_ = 2.74 mM^–1^ s^–1^ for MnCl_2_·4H_2_O, Figure S8L,M). These observations indicate that (i) the removal of Mn-free side products significantly affects the magnetic properties of the material, and *(*ii) confinement of Mn centers within the carbon-dot framework enhances their relaxometric efficiency compared with freely solvated Mn^2+^ ions, likely due to modified water accessibility and rotational dynamics. The stability of the pMnCNDs ([Mn^2+^] = 1.5 mM) was assessed by measuring their relaxivity under different conditions at 1.5 T. In aqueous solution and in cellular medium, the relaxivity values remained unchanged for 1 week, indicating good intrinsic stability in these conditions (Figures 5C and S8). Likewise, the system was stable in the presence of zinc chloride (ZnCl_2_), selected because Zn^2+^ is an endogenous ion, present at relatively high concentrations in blood.^56^ This indicates that Mn ions in this system are not prone to transmetalation processes, and this is a relevant indication for the following *in vivo* studies (Figure S8B). However, the same assay performed in plasma showed a decrease of relaxivity of approximately 30–40% during the first 48 h; thereafter, no further significant variations were detected up to day 14 (Figure S8F,G). Such a temporal profile is consistent with a rapid initial biofluid-induced reorganization at the nanoparticle surface. This is very commonly ascribed to early plasma protein adsorption and/or protein corona formation, causing changes in hydration or water-exchange dynamics, followed by stabilization of that interfacial state. Such effects are known to influence relaxivity in nanoparticle systems, including CNDs.^57^ However, despite this initial reduction, contrast enhancement remains detectable *in vivo* (see *in vivo* studies described below). Finally, pMnCND relaxivities were measured in NH_4_^+^HCOO^–^ buffer over a wide pH range (2.8, 4.2, 6.2, 7.4, and 9.7) for 1 week (Figures 5D and S8C, respectively). The material was again stable under all conditions except in a strong acidic environment (pH = 2.8), and a slight increase of the relaxation constants was detected, suggesting that a fraction of Mn might be released under acidic conditions.

**Figure 5:**
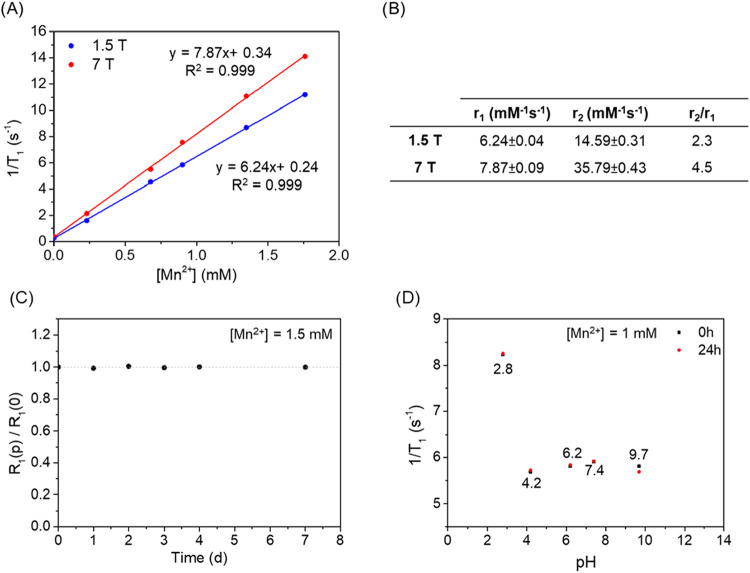
(A) Relaxivity r_1_ of pMnCNDs at 1.5 and 7 T. (B) Relaxivity values and ratio r_2_/r_1_ at 1.5 and 7 T. (C) Evolution of the relative water proton paramagnetic longitudinal relaxation rate R_1_p­(t)/R_1_p­(0) vs time for pMnCNDs (1.5 T). (D) Stability of pMnCNDs at pH 2.8, 4.2, 6.2, 7.4, and 9.7 in NH_4_^+^HCOO^–^ buffer after 24 h. The values remained unchanged for 1 week (1.5 T).

However, the observed stability within the pH 4.2–9.7 range (including physiological conditions) or in the presence of a biologically relevant ion, such as Zn^2+^, together with the good relaxivity properties recorded, supports the robustness of pMnCNDs for biomedical applications.

### *In Vitro* Studies

Studies to assess the behavior of pMnCNDs were performed not only as a due evaluation prior *in vivo* experiment but also because magnetic nanoparticles with additional intracellular functions widen the range of their possible bioapplications.^58^ Cytotoxicity of pMnCNDs was studied by MTT assay using MDA-MB-231 and A549 cell lines (human breast and lung cancer cells, respectively) (Figure 6A). Cell viability was not affected by treatment for 24 h, using up to 200 μg·mL^–1^ of compound, and only a minor effect (cell survival above 70%) was observed at rather high concentrations (500–800 μg·mL^–1^). A similar behavior was observed in HDFa cell line (human dermal fibroblast (adult), Figure S9A) employed as a model of a noncancerous cell line. In this case, the cell viability remained above 80% up to 600 μg·mL^–1^ (the highest concentration employed) at both 24 and 72 h incubation times, confirming the generally low cytotoxicity profile of these materials. Metal internalization into cells was confirmed by ICP-MS analysis of MDA-MB-231 cell pellets recovered after previous treatment with pMnCNDs (200 μg·mL^–1^) for 24 h. Figure 6B shows that Mn accumulated in the cells was ∼2.8-fold higher compared to the control sample (untreated cells carrying endogenous Mn). A control ICP-MS experiment was performed to compare Mn uptake from pMnCNDs and free MnCl_2_ (10 μg·mL^–1^, see Figure S9B). The ICP-MS quantification showed higher intracellular Mn levels in cells treated with pMnCNDs than in those exposed to free Mn^2+^ at equivalent Mn concentrations. This likely arises from the differences in cellular uptake mechanisms. In fact, free Mn^2+^ ions are mainly internalized through ion transport pathways that are tightly regulated by the cell,^59^ limiting their intracellular accumulation. In contrast, CND-associated Mn can be internalized through nanoparticle uptake pathways,^60^ including endocytic processes (as those involved in the uptake of pMnCND, see Methods/Experimental Section below), promoting more efficient cellular accumulation and retention.

**Figure 6:**
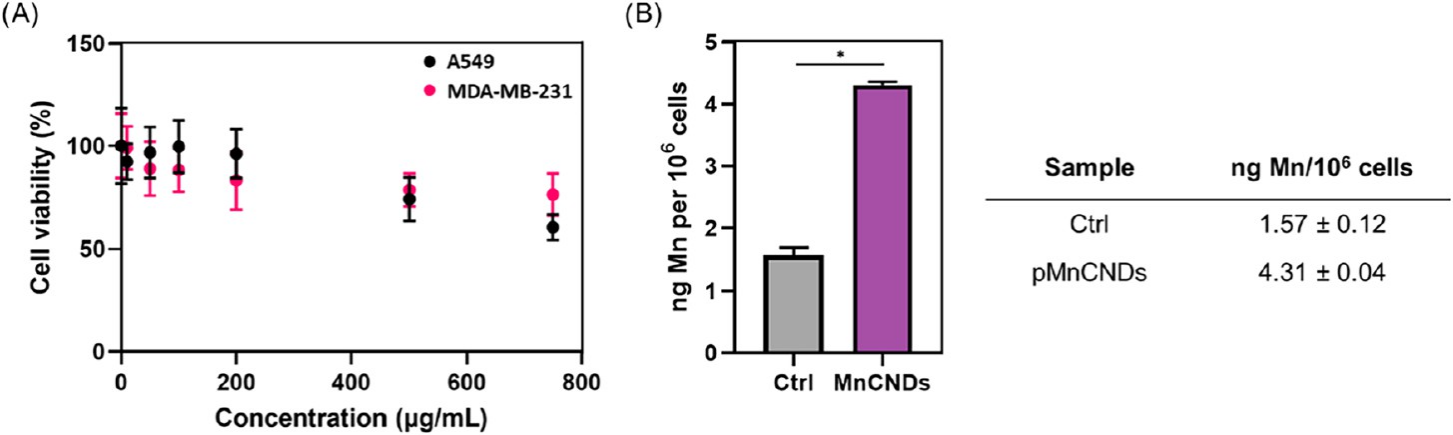
(A) Cell viability (by MTT assay) of A549 and MDA-MB-231 cell lines treated with pMnCNDs for 24 h; (B) ICP-MS analysis of MDA-MB-231 cells treated with pMnCNDs (200 μg·mL^–1^) and untreated cells (Ctrl) (concentration of Mn measured by ICP-Ms is reported in ng per million of cells; * is *P* ≤ 0.01, calculated by *t* test).

It is widely reported that the intrinsic fluorescence of the organic framework of CDs, with suitable QY values, can be employed as an additional optical-based detection mode, both *in vitro* and *in vivo*.^53^ Therefore, the cellular uptake of pMnCNDs was also monitored using confocal laser scanning microscopy (CLSM). CLSM images of A549 cells treated with the material (200 μg·mL^–1^ for 24 h) showed increased fluorescence upon excitation at 405 nm (LP 420 nm) compared to the autofluorescence signal derived from untreated cells used as a control, confirming that the emission of the NPs is a valid tool for optical detection. (Figure S9C). In Figure 7, A549 cells were treated with pMnCNDs (200 μg·mL^–1^) for 24 h, followed by cell membrane staining with CellMask Deep Red (Invitrogen). In CLSM images, the blue emissive material appears localized in the cytosol with poor overlapping with the red emission originating from membrane staining.

**Figure 7:**
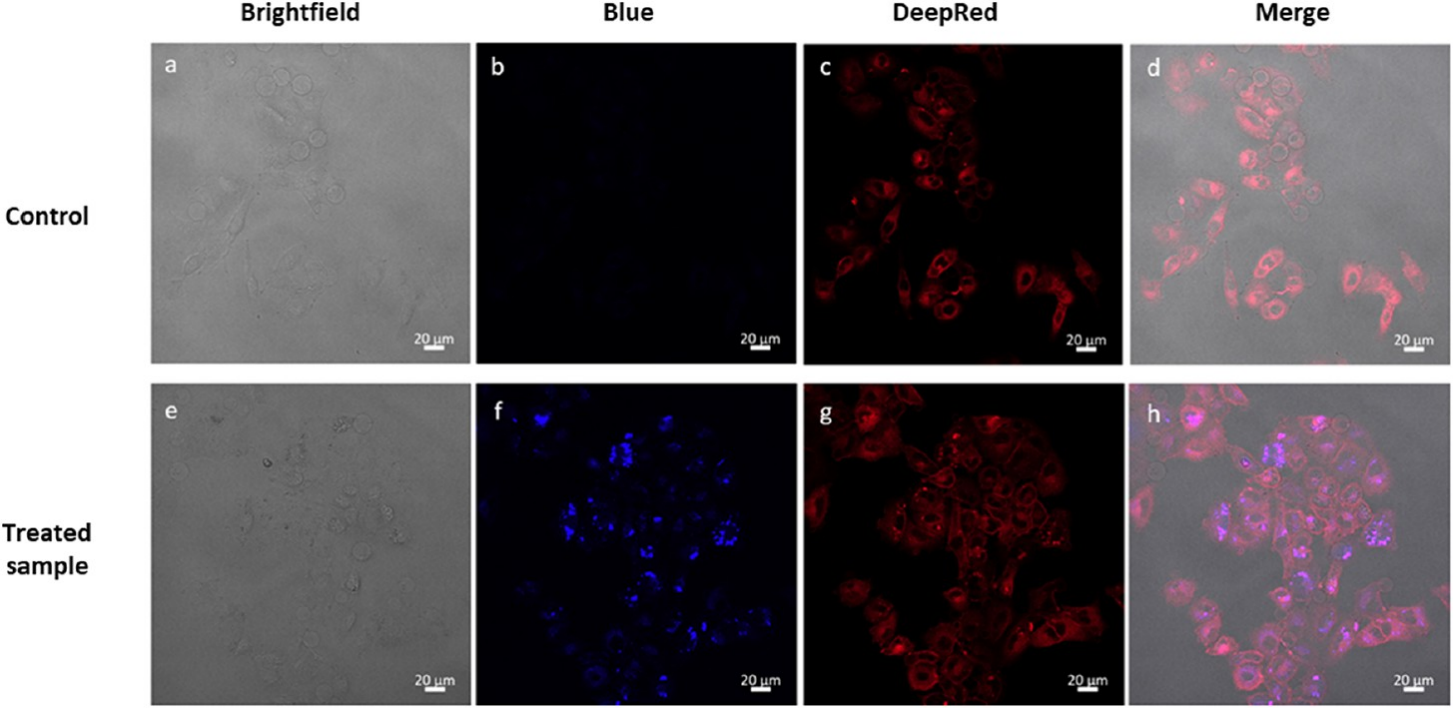
CLSM images (20× air) of fixed A549 cells treated with 200 μg·mL^–1^ of pMnCNDs for 24 h. From left to right: (a) and (e) bright field (BF); (b) and (f) blue emission (λ_ex_ = 405 nm/BP 400–555 nm); (c) and (g) red emission (λ_ex_ = 405 nm/BP 555–700 nm); (d) and (h) merged images of blue+red+BF of nontreated (a–d) and treated (e, f) A549 cells stained with CellMask Deep Red.

Z-stack images of cells were collected using the same samples, and Figure 8 shows representative images of a 20 μm section divided into 11 stacks (only z images 4 to 9 are shown). The higher emission intensity corresponding to pMnCNDs was detected mainly in the central stacks (the highest in z 5/11), confirming cellular internalization (as expected for many CDs). In flow cytometry studies, A549 cells were incubated with different concentrations of pMnCNDs (50, 100, and 200 μg/mL for 24 h) and the analysis showed that cell distribution peaks shifted to highest fluorescence intensities as the material concentration increased, indicating that the cellular uptake of the material was concentration-dependent (Figure 9A, analysis performed using the VioGreen channel, λ_ex_ = 488 nm/BP 500–525 nm).

**Figure 8:**
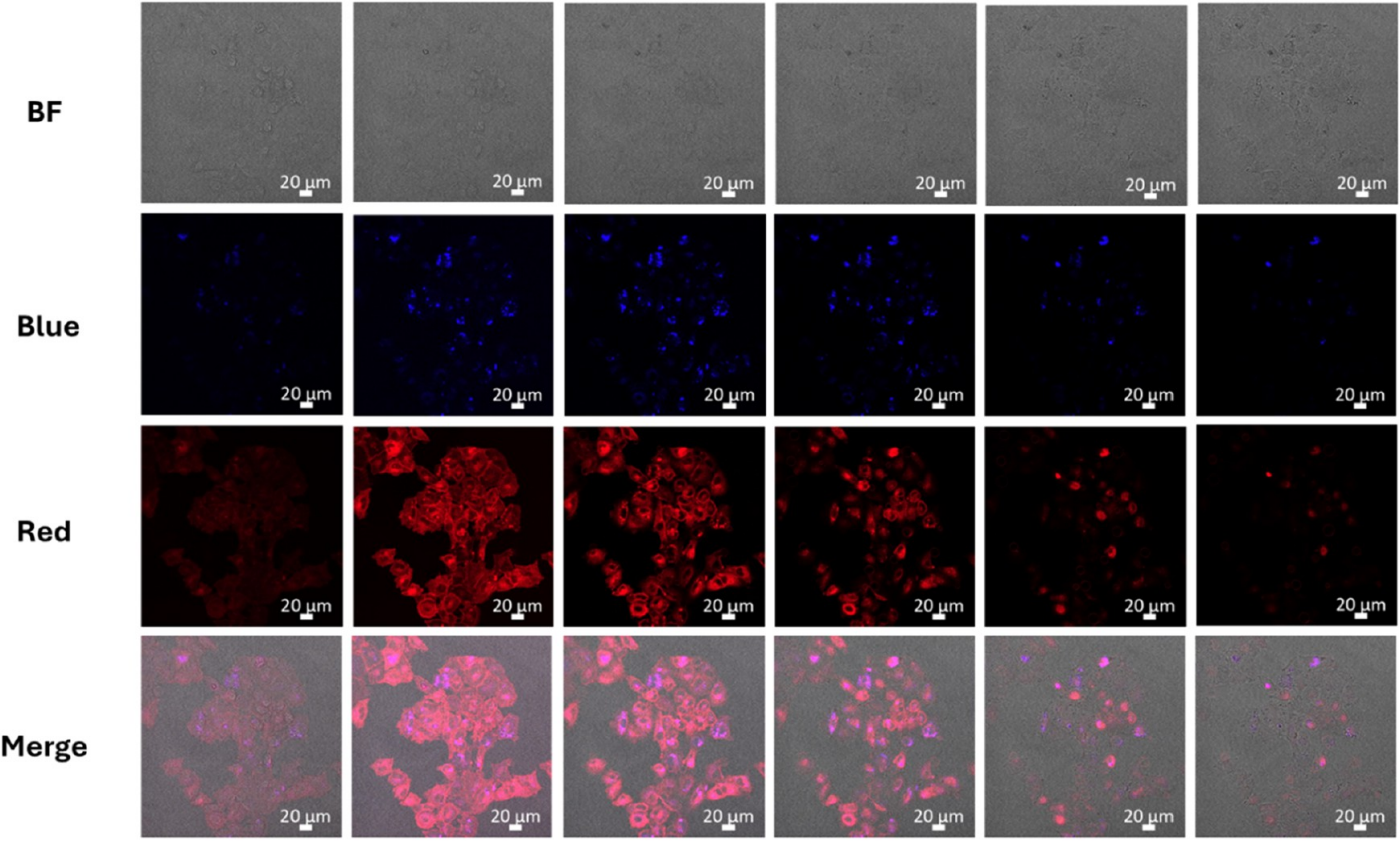
Representative Z-stack Z-stack of CLSM images (20× air) of fixed A549 cells treated with 200 μg·mL^–1^ of pMnCNDs for 24 h followed by staining with CellMask Deep Red (Invitrogen). From left to right: z 4/11 to z 9/11. From top to bottom: bright field (BF), blue emission (λ_ex_ = 405 nm/BP 400–555 nm), red emission (λ_ex_ = 405 nm/BP 555–700 nm), and merged images of blue+red+BF.

**Figure 9:**
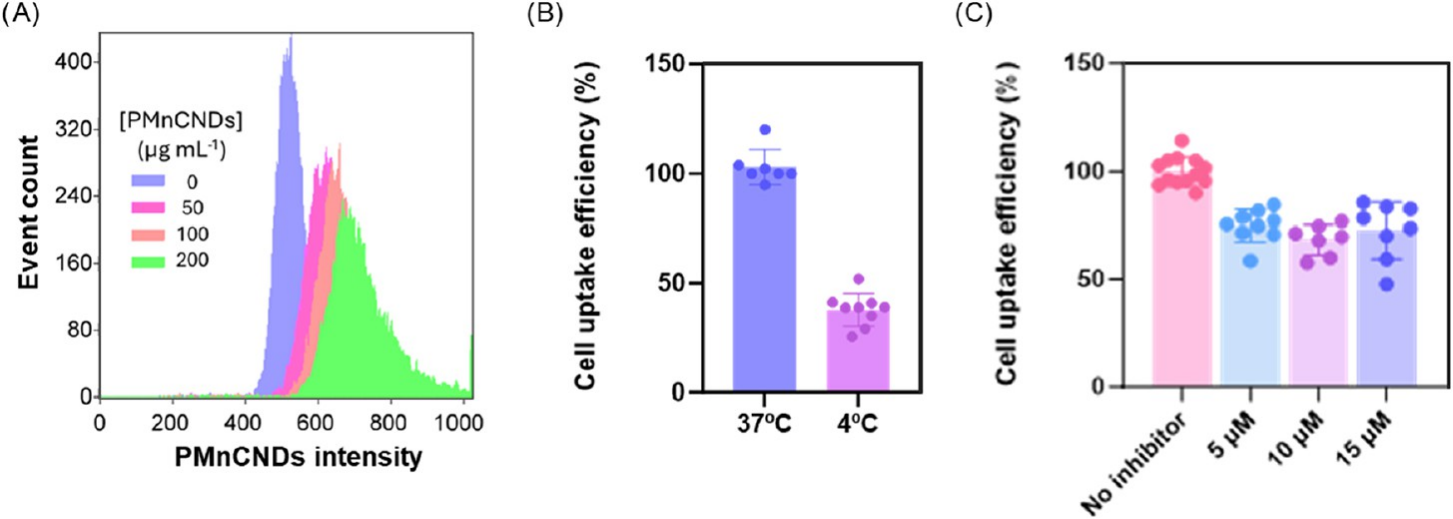
(A) Flow cytometry (λ_ex_ = 488 nm/BP 500–525 nm) analysis of A549 cells treated with different concentrations of pMnCNDs. (B) Cell (A549) uptake efficiency of pMnCNDs upon incubation at 37 and 4 °C for 2 h, and (C) in the presence of cytochalasin (preincubation of cells with the inhibitor for 30 min at 37 °C followed by coincubation with pMnCNDs), in which the concentration of pMnCNDs employed was 200 μg·mL^–1^ and mean fluorescence intensities were measured by flow. In both parts (B) and (C), the results are presented as the mean ± SD of three independent experiments, each performed in triplicate. Statistical analysis was performed using the *t* test. In part (C), each of the treated groups was compared with the no-inhibitor group (****P < 0.0001).

The evaluation of cellular internalization mechanisms of nanoparticles is relevant for several reasons: it is related to the way biological systems respond to biomaterials and how possible therapeutic effects and other functions (i.e., bioimaging) are being delivered;^61^ particularly, understanding the cellular uptake of new CAs for MRI is needed to uncover possible adverse effects as well as potential biotargeting and feasible applications.^62,63^ At the same time, the CND family includes an extraordinary variety of systems with unlimited combinations of morphology, size, synthetic conditions, physical and chemical properties, and each of these combinations may have a different effect on cellular uptake. In most of the studies reported, CNDs penetrate cells through endocytosis or other types of energy-dependent active transport,^64,65^ although uptake through passive diffusion has been observed or intentionally engineered in a few cases.^66,67^ Here, we performed preliminary assays to evaluate which mechanisms are involved in the case of pMnCNDs. First, we compared the penetration of the material in A549 cells under normal physiological temperature and at 4 °C. Figure 9B shows that the accumulation of the material in cells decreases by ∼60% when the incubation is carried out at lower temperatures, which is a clear indication that membrane permeation requires energy-dependent mechanisms such as endocytosis. However, a lower percentage of material also penetrates at 4 °C, implying that temperature-independent mechanisms, such as passive diffusion, may contribute. This is consistent with former reports indicating that reduced-size particles, like CNDs, are more likely involved in passive diffusion uptake.^68,68^

To confirm the contribution of energy-dependent mechanisms, pMnCND uptake into A549 cells was studied in the presence of different known inhibitors. We first selected cytochalasin D, which is known to interfere with the polymerization of actin filaments, and it is implicated in endosome trafficking along the cytoskeleton. In particular, cytochalasin D affects micropinocytosis and phagocytosis, among the processes relying on actin performance; overall, it is reported to be an inhibitor of multiple off-target pathways.^69,70^ The incubation of pMnCNDs in the presence of cytochalasin D (at 37 °C) reduced the uptake of the material by ∼30%, suggesting that actin-dependent endocytosis should occur (Figure 9C). Analogous studies were performed in the presence of other inhibitors, such as amiloride, methyl-β-cyclodextrin (MβC), and chloroquine, whose main activities are summarized in Table 1, and the results are shown in Figure 10. As a first control, the potential cytotoxicity of all inhibitors was initially assessed in A549 cells by MTT assay (Figure S12). No significant cytotoxic effect was observed upon treating cells with different concentrations of inhibitors (up to 1000 μM), indicating that any inhibition of particle uptake observed in this study is unlikely due to compromised cell viability. Amiloride acts by inhibiting Na^+^/H^+^ exchange, leading to an excess of H^+^ production and impairing micropinocytosis, a pathway that is primarily dependent on actin.^71^ However, the coincubation with amiloride did not affect the cellular uptake efficiency of pMnCNDs in A549 cells. Similar results were obtained using chloroquine, a clathrin-dependent endocytosis inhibitor. In contrast, coincubation with MβC partially minimized the trafficking of pMnCNDs in A549 cells by ∼30%. Therefore, caveolae-related and other cholesterol-dependent endocytic mechanisms may contribute to the internalization of pMnCNDs, which is consistent with the accumulation of the material around the nucleus, as observed in CLSM images (Figure 7). In fact, caveolin-dependent endocytosis transports the cargo mainly to the Golgi apparatus and endoplasmic reticulum, surrounding the nuclear area.

**Table 1:** Inhibitors Employed in Our Study and Relative Interference with Endocytosis

inhibitor	affected pathway	mechanism
cytochalasin D^72^	multiple pathways	actin polymerization inhibitor
amiloride^73^	macropinocytosis	inhibition of Na^+^/H^+^ exchange
methyl-β-cyclodextrin^69^	caveolin-dependent endocytosis, lipid raft-mediated endocytosis	cholesterol depletion
chloroquine^74^	clathrin-dependent endocytosis	suppression of PICALM expression (phosphatidylinositol binding clathrin assembly protein)

**Figure 10:**
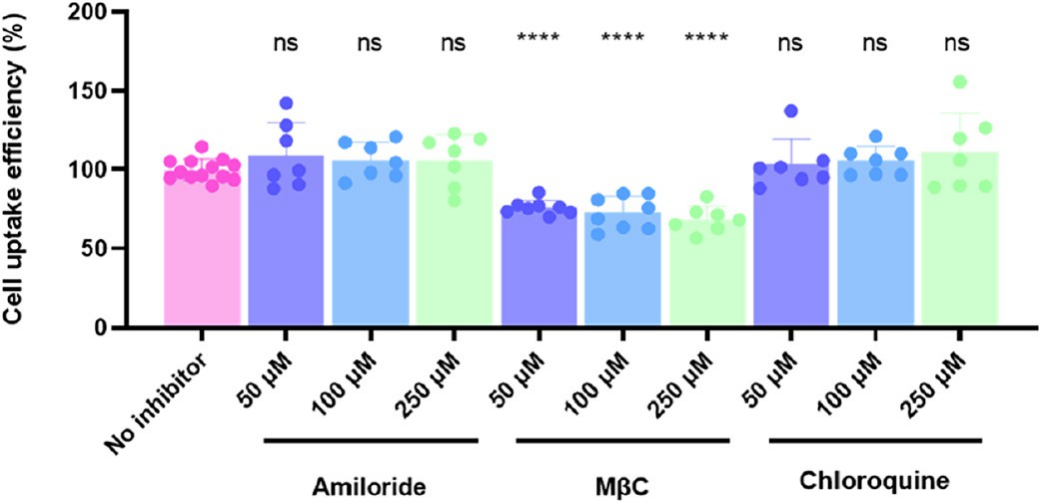
Cell (A549) uptake efficiency of pMnCNDs (200 μg·mL^–1^) after pretreatment with amiloride, M-β-C, and chloroquine (each inhibitor was used at three different concentrations). Statistical analysis was performed using the *t* test. Each of the treated groups was compared to the no-inhibitor group; ns indicates no significant difference, whereas **** is *P* < 0.0001.

The partial inhibition observed after treatment with cytochalasin D and MβCD indicates that pMnCND internalization is not mediated exclusively by actin-dependent or caveolin-dependent pathways. This is consistent with the possible passive internalization observed in Figure 9B. Altogether, these results suggest the concurrent involvement of multiple internalization routes. This behavior is consistent with previous reports showing that particles below ∼10 nm lie at the boundary between nanomaterials and macromolecules, and therefore typically enter cells through a combination of pathways rather than a unique mechanism.^69^

It should be noted that the present study was conducted in A549 cells as a representative of an epithelial cancer model. Endocytic processes depend strongly on membrane composition and protein expression patterns, which vary across cell types; consequently, the relative contributions of individual uptake pathways may differ in other cellular systems. Here, we aimed to establish the uptake behavior and biological performance of pMnCNDs in a widely used cancer cell line, and therefore, the conclusions should be interpreted within this specific cellular context. Nevertheless, the results obtained clearly indicate that this class of carbon dots undergoes predominantly endocytosis-mediated internalization. This constitutes fundamental yet valuable information, as it provides a basis for future biotargeting design strategies.

### *In Vivo* Imaging by MRI

To validate pMnCNDs as MRI CA, biodistribution studies were conducted using C57BL/6JRj male mice (*n* = 3 for each experiment). Doses of pMnCNDs (0.075 mmol Mn·kg^1–^) were prepared in PBS 20 mM, and the pH was adjusted to 7.3 with NaOH 3 M before administration. Initially, MRI images were acquired from the mice before injection to establish a basal image. After that, pMnCNDs were administered intravenously via the tail vein, and MRI scans were repeated 1 and 24 h postinjection. After, mice were sacrificed, and organs were harvested for further *ex vivo* analysis. The MRI images are shown in Figure 11A, where significant shortening of T_1_ relaxation times was evident in the liver, kidney, and bladder at 1 h postinjection, indicating the presence of the material in these organs. T_1_ values predominantly recover within 24 h in these main organs, suggesting that a typical hepatobilliary-renal clearance is occurring (analysis in Figure 11B). T_2_ values were also monitored: minor changes with respect to the basal signals were observed in the same organs, although pMnCNDs are less efficient as T_2_ CA (see Figure S13). In *ex vivo* analysis, key organs were excised, dried, digested, and analyzed by ICP-MS, finding rather low levels of possible residual Mn (around 0.2% in most organs and 1.2% in kidneys). The biodistribution we observed for pMnCNDs is analogous to that reported for other low-dimensional carbon-based nanomaterials^36,75^ and many NPs.^76^ However, during the biodistribution study, we observed some accumulation of material in the salivary glands, with MR contrast persistent after 24 h, in this region only. To investigate this further, the biodistribution study was repeated, but the animals were monitored for 6 days after injection, confirming the behavior described above in all organs. Focusing on the salivary glands, the levels of Mn increased by 3.3 folds in 24 h compared to control, while the recovery of basal T_1_ values and Mn levels (in *in vivo* and *ex vivo* experiments, respectively) were slower. In fact, only on day 6, ca. 50% of the signal was recovered in MRI scans, while the levels of Mn in excised glands returned close to basal (Figure 12). This suggests that the elimination of the Mn agent from this region is not as fast as in other organs. We observed that high fractions of Mn were eliminated through urine (i.e., 22% of the injected metal was found in the urine sample collected after 90 min (Figure S14) and feces (Figure S15)), but accumulation and excretion through these pathways is faster, while saliva might be an additional but slower excretion route. Based on previous reports, as well as our experience with *in vivo* studies of analogous metal-based CNDs, the observed accumulation of the material in salivary glands was unexpected. In general, excretion of drugs through saliva is not a primary route but a known alternative to hepatobiliary and renal clearance, and this can be employed as drug screening method in few cases.^77^ Some small drugs, with low protein binding capability, are transferred from plasma to saliva through passive diffusion; however, the topic is poorly reported in the case of small NPs, and whether this might or not involve specific receptors, is still unclear.^78,79^ Securing an explanation for the observed accumulation of pMnCNDs in salivary glands was beyond the scope of this work, but this finding eventually opens up new possible applications for this material, in fields such as drug delivery, theranostics, and antimicrobial therapies.

**Figure 11:**
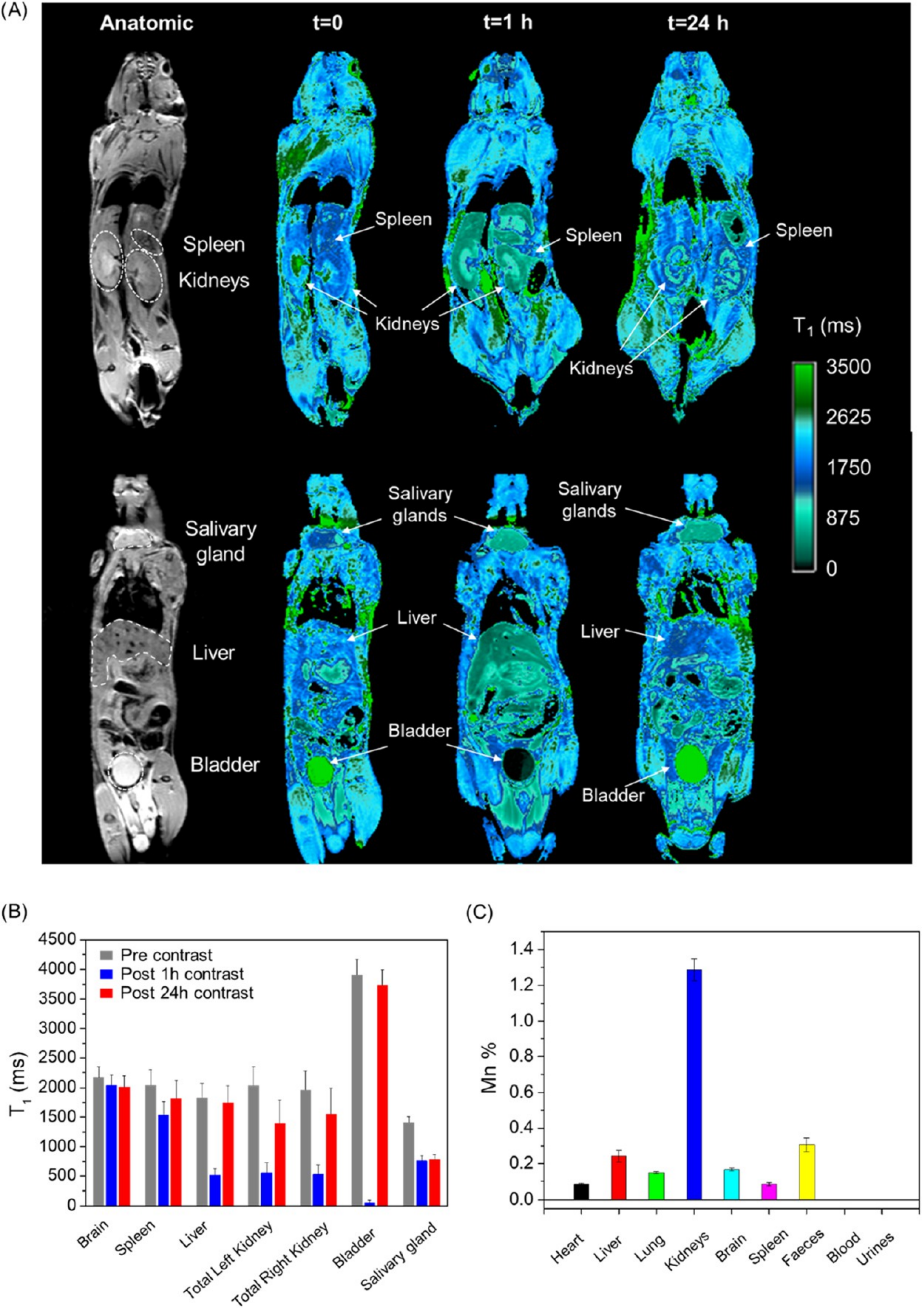
(A) Anatomical image (left) and T_1_ relaxation maps (water-green color) of selected MRI images of mice after injection of pMnCNDs (0.075 mmol Mn·kg^1–^). (B) T_1_ relaxation values of the analyzed organs before and after the administration of pMnCNDs (*n* = 3; results are the mean ± SD). (C) *Ex vivo* analysis: Mn level measured by ICP-MS analysis of excised (after 24 h) and dried organs. The chart shows the percentage of Mn relative to the mass of each organ (%w/w, mean ± SD of *n* = 3) after subtraction of possible endogenous Mn.

**Figure 12:**
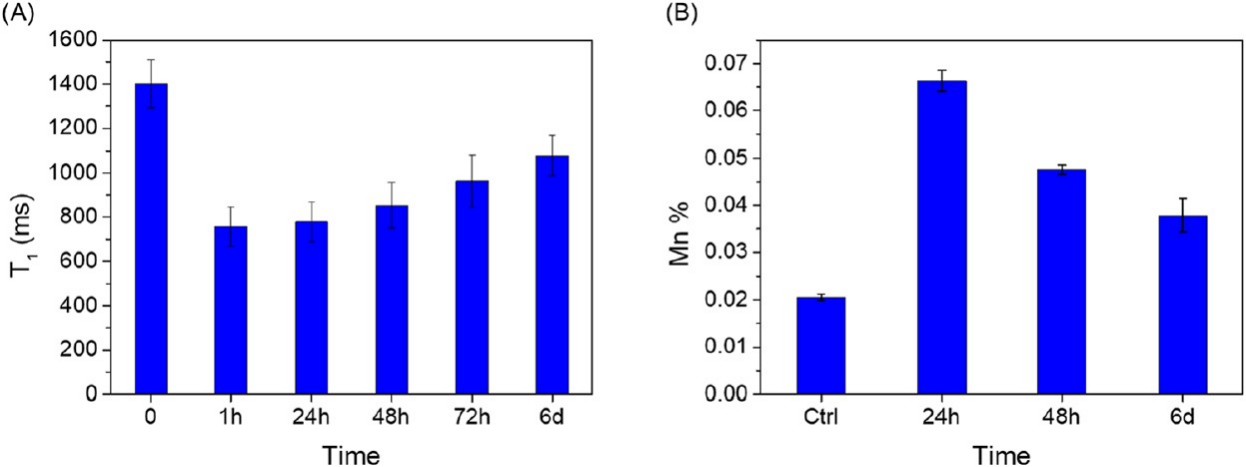
(A) T_1_ relaxation values of the salivary gland before and after administration of pMnCNDs (*n* = 3; mean ± SD). (B) Mn% in the salivary glands before (Ctrl) and after administration for 6 days.

The biocompatibility of the material was further evaluated by histological analysis of key organs excised 24 h, 48 h, and 6 days postinjection (Figure 13). No tissue damage was observed in any of the organs examined, including the kidneys, liver, and salivary glands, where the material was mostly accumulated, confirming that pMnCNDs do not cause adverse effects in these tissues *in vivo* in the short- and medium-term.

**Figure 13:**
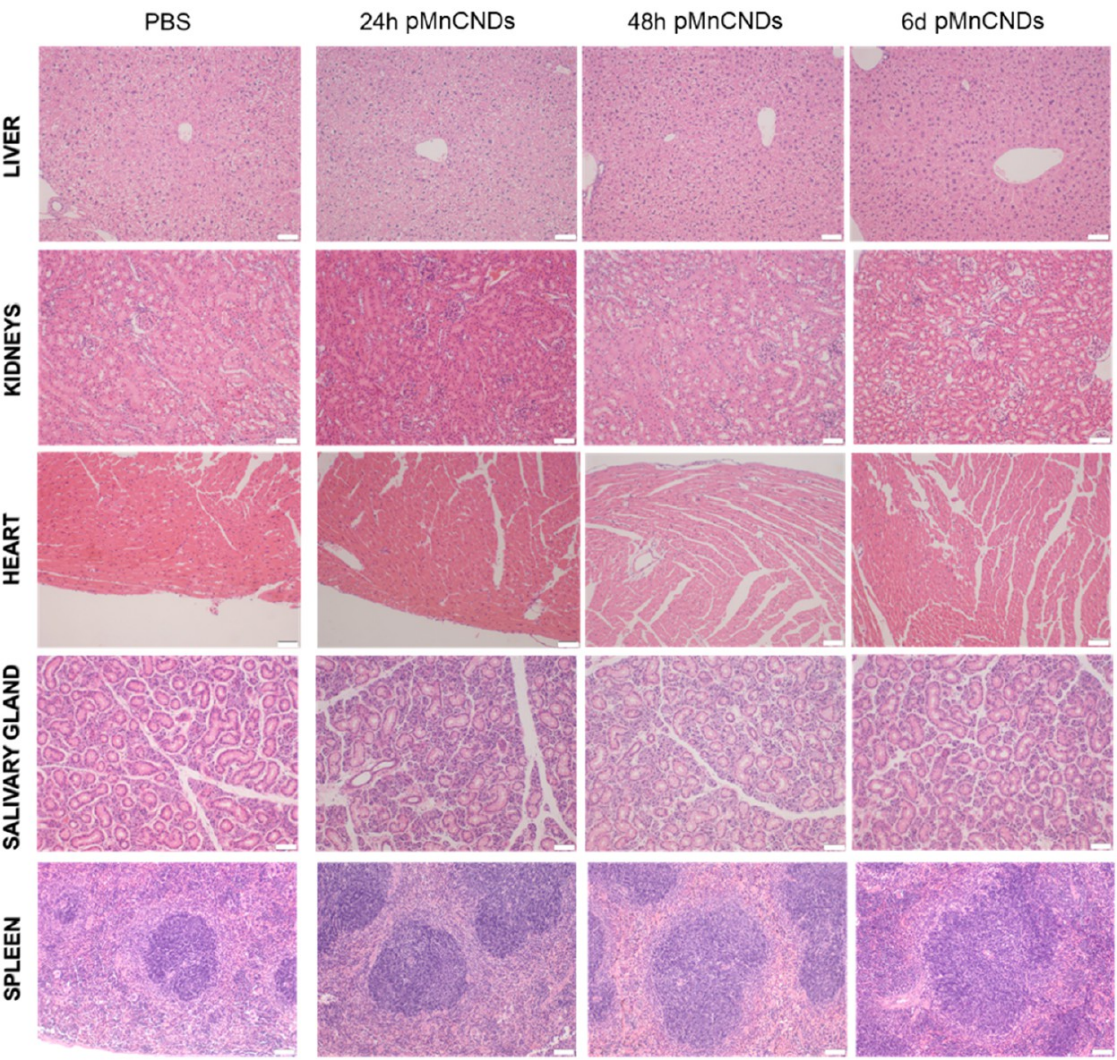
*Ex vivo* H&E 10× images of the liver, kidneys, heart, salivary glands, and spleen 24 h post injection of PBS (control, left) and after 24 h, 48 h, and 6 d of pMnCNDs (0.075 mmol·kg^1–^). The scale bar is 50 μm.

Finally, we performed a long-term *in vivo* toxicity evaluation by monitoring key renal and hepatic biomarkers to assess potential alterations in the organs where the materials predominantly accumulated. pMnCNDs were administered intravenously to C57BL/6JRj (*n* = 9, 8 weeks old), and biological samples (blood and urine) were collected at four time points: day 0 (baseline, prior to administration), day 6, day 18, and day 28 postadministration. Serum biomarkers included creatinine and urea (renal function), alanine aminotransferase (ALT/GPT), and aspartate aminotransferase (AST/GOT) (hepatic function), as well as amylase. In urine samples, the total protein was quantified as an additional indicator of renal integrity. Throughout the study period, all animals remained in good health with normal grooming, feeding behavior, and activity levels. Body weight (Figure 14A) increased progressively throughout the course of the experiment, consistent with the expected growth for mice of this age and strain.

**Figure 14:**
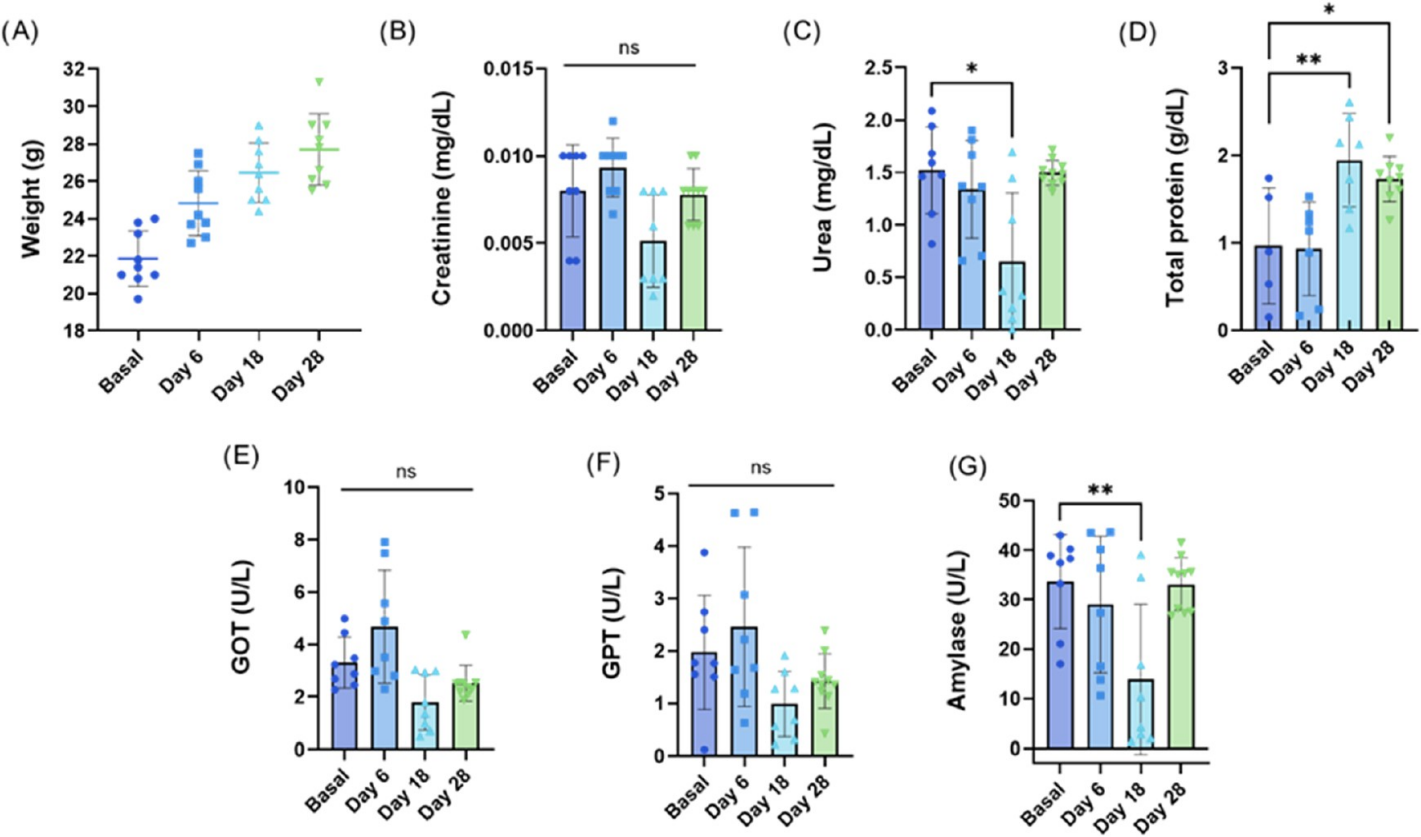
Long-term *in vivo* toxicity evaluation. All graphs refer to mice (*n* = 9) treated with pMnCNDs ([Mn^2+^] = 0.075 mg·mL^–1^) during the long-term toxicity experiment. (A) Body weight variations; (B) serum creatinine concentrations; (C) urea concentration in urine; (D) total urinary protein; (E), (F), and (G) serum GOT, GPT, and amylase concentrations, respectively (statistical significance was defined as *p < 0.05; **p < 0.01; ***p < 0.001; ****p < 0.0001; ns, not significant).

Serum indicators of renal function remained stable across all of the time points. Creatinine levels (Figure B) were consistently low and did not deviate meaningfully from baseline values, indicating preserved glomerular filtration. Urea concentrations (Figure 14C) decreased slightly on days 6 and 18, returning to basal levels by day 28 with no indication of renal impairment. These small fluctuations may reflect normal biological variability, hydration status, or minor procedural differences during sample collection rather than true toxic effects.

Urinary protein levels, reported in Figure 14D, remained comparable to baseline on day 6 but showed a transient increase on day 18, followed by partial normalization on day 28. Since this rise occurred in the absence of changes in serum creatinine or urea, it likely reflects a mild and transient tubular response rather than glomerular dysfunction.^78,79^

Liver enzyme activity remained within the physiological range throughout the study following pMnCND administration. Both GOP and GPT (Figure 14E,F) showed a negligible rise on day 6, followed by a decrease below baseline by day 18 and a return toward initial values by day 28. These variations were small and did not follow a pattern indicative of a hepatic injury. These fluctuations are consistent with normal physiological variability or small differences in sampling procedures and do not reveal hepatic injury. Overall, the enzyme profiles suggest that pMnCNDs did not induce measurable liver toxicity.

Since pMnCNDs were found to accumulate in salivary glands, amylase levels (Figure 14G) were monitored as an indirect functional indicator. Amylase activity decreased on days 6 and 18, with a near-complete recovery by day 28. The transient reduction, together with full recovery, suggests that any early effect on the salivary gland function was temporary. Minor variations may also reflect physiological differences or sampling conditions rather than true toxicities.

Across all evaluated biochemical parameters and physiological observations, no evidence of systemic or organ-specific toxicity was detected within the 28-day observation period. Combined with stable body weight and the absence of behavioral or clinical signs, these findings support good short-to-midterm tolerability of pMnCNDs under the administered dose and experimental conditions. Overall, the results indicate a favorable biocompatibility profile within the evaluated time frame. The minor fluctuations observed in selected parameters were transient and nonprogressive and can be attributed to normal biological and procedural variability.

## Conclusion

Here, we employed the technology of CNDs to develop luminescent carbon-based NPs loaded with Mn­(II) (pMnCNDs). Despite CNDs having gained huge popularity in the last few decades, important debates continue regarding controls over synthetic procedures and, most importantly, improving purification protocols. Here, we proved that a careful selection of starting precursors can indeed improve synthetic yields, and, most importantly, dialysis should not be accepted as a universal purification method valid for all CNDs. In some cases, as reported here, additional purifications are essential to achieve more reliable and homogeneous formulations. The new MnCNDs obtained present an amorphous structure and a negative charge, being desirable for CA applications (reduced risk of toxicity). pMnCNDs contain 5% w/w Mn, stabilized in oxidation state +2 (as required for an efficient and safe MbCA). They present an average size of 7 nm, assessed by AFM and STEM, while TEM and STEM-EDX revealed a possible Mn-dense core of about 4 nm. This type of data is relevant to prove an effective colocalization between the carbon framework and the metal, and they are rarely exhibited in other reports. The relaxivity (r_1_) values of pMnCNDs at 1.5 and 7 T are comparable to those of GbCAs, and, importantly, they are stable over time in water, across different pH conditions, and are susceptible to transmetalation, these factors often limiting the advancement of many MbCAs.

pMnCNDs do not show relevant cytotoxicity in different cell lines, up to 500 μg·mL^–1^; they are cell permeable (as confirmed by ICP-MS analysis) and with blue emission intensity suitable for detection in cells by FI, using both CLSM and flow cytometry. CLSM studies indicate that the material accumulates in the cytosol around the nucleus region, and first cell uptake experiments suggest that internalization by passive diffusion and endocytosis are both occurring.

Finally, and most importantly, *in vivo* investigations confirmed that pMnCNDs behave as promising MRI contrast agents, showing predictable biodistribution and efficient elimination through hepatic and renal pathways as well as potential excretion through salivary glands. This occurred without the necessity of biotargeting activity, meaning that the material is well-detectable by MRI without specific tissue accumulations. Additionally, the absence of tissue damage observed in the histological analysis or relevant long-term (4 weeks) toxicity supports the biocompatibility of the pMnCNDs. One important aspect of these materials concerns the reliability of the observed MRI contrast. The SEC-HPLC purification strategy, together with the physicochemical characterization (particularly relaxometric studies, AFM, and TEM/STEM-EDX analyses), strongly suggests that free Mn ions or low-molecular-weight Mn-containing side products (if present) were largely removed and that the MRI performance predominantly arises from Mn associated with the nanoparticle framework. This interpretation is further supported by the *in vivo* biodistribution profile, which showed accumulation mainly in the liver, spleen, and kidneys, without marked enhancement in highly perfused tissues typically associated with freely diffusible Mn species. Further studies will focus on combining this system with additional functionalities, both by postsynthetic conjugation with biologically relevant moieties (i.e., to target tumors or other pathologies) and by tuning the emission profile of the carbonaceous core toward the highest wavelengths (i.e., NIR).

## Methods/Experimental Section

An extensive Experimental Section can be found in the SI. All animal studies were performed at CICbiomaGUNE. The animals were maintained and handled following the Guidelines for Accommodation and Care of Animals (European Convention for the Protection of Vertebrate Animals Used for Experimental and Other Scientific Purposes). All animal procedures were performed under the European Union Animal Directive (2010/63/EU). Experimental procedures were approved by the local Ethical Committee of CIC biomaGUNE and by the local authorities (Diputación Foral de Guipúzcoa, project number PRO-AE-SS-225).

## Supplementary Material



## Notes

Detailed experimental procedures for the synthesis and purification of the MnCNDs, descriptions of the analytical methods and instrumentation used, and comprehensive information on the physicochemical characterization, relaxivity measurements, and *in vitro* and *in vivo* studies + supporting figures (PDF)
