# Supplementary material for: Manganese-Doped Carbon Nanodots as Next-Generation MRI and Fluorescence Imaging Agents
Source: ACS Nano. 2026 Jul 20;20(30):21137–54. doi: 10.1021/acsnano.6c03209 (PMC13450473; doi:10.1021/acsnano.6c03209)
Supplement: Supplementary file 1 [file nn6c03209_si_001.pdf]

Supporting Information

Manganese-Doped Carbon Nanodots as Next-Generation MRI and Fluorescence Imaging Agents

Michele Cesco,[1,2] Lydia Martínez-Parra,[1] Samantha Marcelino Apresto,[1,2] Cecilia Wetzl,[1] Begoña Maria Echeverría-Beistegui,[1] Jesús Ruiz-Cabello,[1,3,4,5] Lucia Cardo*[1] and Maurizio Prato*[1,3,4,6]

[1]Center for Cooperative Research in Biomaterials (CIC biomaGUNE), Basque Research and Technology Alliance (BRTA), Paseo de Miramón 194, Donostia-San Sebastián, 20014, Spain.
[2]University of the Basque Country UPV-EHU, Donostia-San Sebastián, 20018, Spain.
[3]Ikerbasque, Basque Foundation for Science, Bilbao, 48013, Spain.
[4]Centro de investigación en red de enfermedades respiratorias (CIBERES). Instituto de Salud
Carlos III, 28029 Madrid, Spain
[5]Departamento de Química en Ciencias Farmacéuticas, Universidad Complutense de Madrid, Madrid, 28040, Spain.
[6]Dipartimento di Scienze Chimiche e Farmaceutiche, Università degli Studi di Trieste, Trieste, 34127, Italy
*corresponding authors: lcardo@cicbiomagune.es, mprato@cicbiomagune.es

Keywords: carbon nanodots (CNDs), manganese-doped nanoparticles, T1-weighted MRI, nanomedicine, nanoprobes for biomedical imaging, manganese contrast agents.

Materials & methods

Microwave assisted synthesis

EDTA (1 mmol), β-Ala (1 mmol), $MnCl_2 \cdot 4H_2O$ (0.25 mmol) and Glutaraldehyde 50% solution water (1 mmol) were mixed with 100 µL of Milli-Q water and 200 µL of HCl 0.1 M. The resulting mixture was heated in a focused microwave (CEM Discover-SP) at 240 °C, 75 W, for 30 min, to achieve a dark orange/brown solution. Upon dilution in Milli-Q water (up to 10 mL), little black precipitate was observed and was eliminated by using a 0.2 µm filter. The obtained solution was dialysed against water for 72 hours. The solution was then freeze dried and stored at room temperature. Reaction yield 15.2% (in mass).

We employed fixed-power microwave (MW) irradiation with temperature control as the operating method. In this instance, a power of 75 W and maximum temperature of 240ºC were chosen as parameters to reach a high pressure (approximately 300 PSI), which is crucial for achieving a high degree of carbonization of the material. The MW reaction profile, shown in Figure S1A, illustrates the power, temperature and pressure changes during the synthesis. For MnCNDs synthesis, 1 mmol of EDTA and 0.25 mmol of $MnCl_2 \cdot 4H_2O$ were mixed in 100 µL of water and 200 µL of HCl 0.1 M. The heterogeneous mixture was stirred for 5 minutes. Then, 1 mmol of β-Alanine and

glutaraldehyde were added to the mixture and stirred for an additional 5 minutes. The reaction initially takes place through chelation of the Mn metal by EDTA, followed by the polymerization between the formed complexes and the other molecules. Glutaraldehyde may react with the amines of the system, forming imine bonds, which may enhance the reactivity of the system during the carbonization process, resulting in a higher yield of the product obtained. After completion of the reaction, the mixture was filtered using a 0.2 μm filter to remove insoluble products, followed by dialysis (MWCO 500-1000Da, 72 h against Milli-Q water) and freeze-drying to obtain crude Mn-doped CNDs (cMnCNDs)., These initial purification steps effectively remove water insoluble products, small molecules and starting materials. Comparing ^{1}H-NMR spectra of the reagents (β-Ala, EDTA and glutaraldehyde) with cMnCNDs (Figure S1B) is possible to see the absence of precursor residues. For sake of clarity, in this experiment the ^{1}H NMR of Na_2EDTA instead of EDTA was employed due to the lack of solubility of this precursor in the NMR solvent (D_2O). In cMnCNDs spectrum, the very wide band between 1 and 4 ppm might indicate presence of material, however the presence of the metal highly disturbs the signal.[1] Proton NMR of glutaraldehyde shows the signals of a mixture of hydrated forms of the carbonylic groups. Gel Electrophoresis (GE) analysis of cMnCNDs reveals four main populations: two positive and two negative bands (Figure S1C). By cutting the gel and analysing them by ICP-MS, it was confirmed that the metal is present only in the negative bands, similar to observations with GdCNDs reported previously.[1] RP-HPLC-C_{18} absorbance chromatogram (λ_{abs}=254 nm) shows a width band representative of a large population distribution (Figure S1D). From 5 to 95% of acetonitrile in water (in 15 min) was the gradient employed for this analysis. The band observed within the 1-8 minutes range, when the eluent is predominantly water, confirms the hydrophilicity of the material. Such a broad signal is consistent with a compound behaving like a material, while molecules would produce sharp peaks.

SEC-HPLC fluorescence and absorbance chromatograms of cMnCNDs (Figure S1D), performed using $NH_4^+HCO_3^-$ at pH 8.5 as buffer, indicate the presence of two main fluorescent populations from 10.8 to 13 and 13 to 14.8 minutes, respectively, and a main absorption band from 10.8 to 13 minutes. Due to our interest in fluorescent dual MRI/FI probes, both fluorescent fractions were collected (Figure S1E) to obtain purified-MnCNDs (pMnCNDs).

GE

Freshly prepared agarose gel (1% w/v agarose in 1x TAE buffer (40 mM Tris Acetate, 1 mM EDTA, pH 8)) were employed for all GE. The analysis was run on a PowerePac™ Basic Power Supply from Biorad (120 V, 25 minutes) in 1x TAE buffer at loading 10 μL of 1 mg/mL solution of the material. The gel was then revealed using a ChemiDoc MP Imaging system from Biorad.

HPLC-SEC

High performance liquid chromatography analysis was performed on a Shimadzu SCL-40, equipped with LC-20AR pumps, CTO-40C column oven, SPD-M40 photo diode array detector and

a RF-20A fluorescence detector. Size exclusion chromatography was performed with a ultrahygrogel 120 (7.8 x 300mm) column at 0.5 mL/min, 30°C and using 50 mM ammonium bicarbonate buffer at pH 8.

Optical spectroscopy

UV-Vis and fluorescence characterizations were performed using a VarianCary 5000 spectrophotometer and a FS5 Spectrofluorometer (Edinburgh Instruments), respectively. The spectrofluorometer include a SC-30 integrating sphere cassette, that was employed to determine quantum yields. In both cases samples were dissolved in Milli-Q water, and quartz cuvettes from Hellma® (10 mm pathlength) were employed.

ICP-MS

Known concentrations of CNDs were dissolved in Nitric Acid (1 mL, 67-69%, Optima™, for Ultra Trace Elemental Analysis) and the digested using a Microwave Digestion System Speedwave XPERT, followed by dilution with nanopure water (10 mL). The amount of Mn was measured by iCAP-Q ICP-MS equipped with collision/reaction cell and Kinetic Energy Discrimination (KED). The calibration curve was prepared from a multi-element stock solution certified in 2% HNO_3 (Inorganic Ventrurs CMS-5, 10 ug/mL Ag, Al, Ca, Co, Cr3, Cs, Cu, Fe, K, Li, Mg, Mn, Na, Ni, Sr, Zn, Lot K2-MEB653508) and subsequently diluted in the same medium to the required concentrations. The limit of detection (LOD) was determined as three times the ratio of white noise to the slope, whereas the limit of quantification (LOQ) was defined as ten times this ratio. Accordingly, for this calibration line, the LOQ was 0.03 µg/L (ppb). The lowest reliably quantifiable concentration within the established linearity corresponded to the lowest calibration point (0.5 µg/mL). Below, a representative calibration curve:

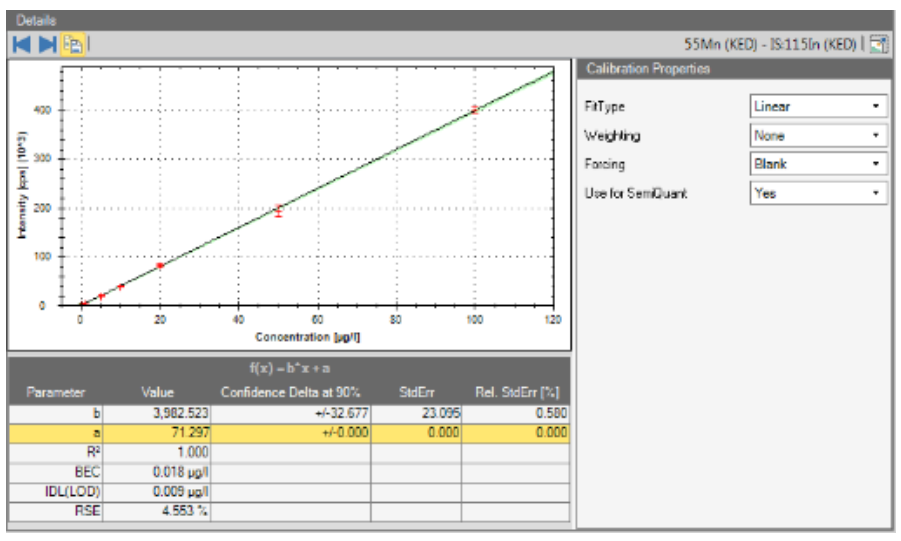

AFM

AFM characterization was carried out with JPK bioAFM system using a tapping mode tip with a frequency of 320 Hz (Bruker TESPA-V2). The nanoparticles were dissolved in water (0.001 mg·mL^{-1}) and spin coated on freshly cleaved mica. Images were processed using WSxM 5.0.

TEM

Samples for TEM were prepared by deposition of 1 μL of material, (dissolved and sonicated in water/methanol=1/1) on top of a copper grid coated with a layer of ultrathin carbon film (01824, TED PELLA, INC.). TEM images were acquired with a JEOL JEM 2100F microscope. Free software ImageJ was used to select particles (>150 from different window views) and measure their size (largest diameter). Frequency distribution of sizes and statistical analyses were created with GraphPad Prism 9.

NMR

All NMR data were collected on a Bruker AVANCE III NMR spectrometer (11.7 T, 500 MHz for ^{1}H). The spectra were processed with MestReNova software version 7.1.1-9649.

XPS

All XPS analysis were gathered using XPS/UPS – SPECS SAGE HR-100, equipped with a 100 mm radius PHOIBOS analyser.

Thermogravimetric Analysis (TGA)

All TGA experiments were performed under nitrogen atmosphere (25 mL·min^{-1} flow rate) using a TGA Discovery (TA Instruments). The procedure used is composed of a first equilibration at 100 °C for 20 min and then heated at a rate of 10 °C·min^{-1}, in the range from 100 to 800 °C.

FT-IR

FT-IR spectra were collected on a Bruker Invenio-X with Attenuated total reflectance (ATR) mode.

pMnCNDs Titration

A 1 mg·mL^{-1} solution of pMnCNDs was prepared in milliQ water. Native pH observed 5.7 was lowered up to 2 by using solution 1 M of HCl. Then, a solution 0.5 M of NaOH was used to gradually bring the pH up to 12.1.

Relaxivity studies

In minispec experiments, relaxation times (T_1 and T_2) were measured for different concentrations of pMnCNDs in water (Milli-Q) a Bruker Minispec MQ60 (Bruker Biospin GmbH) contrast agent analyzer at 1.5 T and 37ºC. For MRI 7T, experiments were performed on a 7 Tesla Bruker Biospec 70/30 USR MRI system (Bruker Biospin GmbH, Ettlingen, Germany), interfaced to an AVANCE III console. A BGA12 imaging gradient (maximum gradient strength 400 mT/m) system with a 40 mm diameter quadrature volume resonator was used for the acquisition. Relaxivity values were calculated (using Origin 9.8) from the curve fitting of $1/T_1$ or $1/T_2$ (s^{-1}) against Mn concentration in samples (mM).

Stability studies

Stability of pMnCNDs were performed by dissolving the material (crude and pure) in Milli-Q water (300 µL, 1.5 mM of Gd measured by ICP-MS) and measuring T_1 and T_2 relaxation times for 1 week (Bruker Minispec MQ60, 1.5 T and 37ºC) at different time points (0, 1, 2, 3, 4, 5, 7 days). Stability experiments at different pH were performed in ammonium formate, to allow the screening of a wide range of pH. 100 mM stock solution of $NH_4^+HCOO^-$ was prepared and divided in different vials. Then, in each vial, pH was adjusted with few microliters of formic acid or ammonium to 2.8, 4.6, 6.2, 7.4 and 9.7. For each solution, PMnCNDs was dissolved and T_1 and T_2 of these final solutions (with 1.5 mM Mn) were measured at times 0 and 24 h.

Cell Biology Studies

A549 (human epithelial cells, lung, carcinoma) were purchased from the Health Protection Agency, European collection of cell cultures (ECACC), whilst all media and media components and buffers were from Merck. Cells were cultivated at 37°C and humidified atmosphere (5% CO_2, 95% air), in RPMI 1640, supplemented with 10% FBS, 1% L-glutamine and 1% Penicillin/Streptomycin. Cells were refreshed every 2-3 days and regularly checked for the absence of contamination.

Cell viability assays

96 well plates were seeded overnight with defined number of cells (4000 per well in 100 µL of media). Cells were treated with triplicates of at least six different concentrations of pMnCNDs, typically from 10 up to 800 µg mL^{-1}, and left in incubator (37°C, 5% CO_2, 95% air) for 24, 48 or 72 h. Cells were then washed with PBS (3x) and treated with 100 µL of 3-(4,5-dimethylthiazolyl-2)-2,5-diphenyltetrazolium bromide (MTT) in medium (final concentration 0.5 mg/mL) for 3 hours. Medium was carefully removed from each well, the purple crystals of formazan dissolved in 200 µL DMSO and absorbance at 550 nm was measured using a microplate reader (GENios Pro, Tecan). Cell viability charts were generated with GraphPad 9.1 employing measurements from at least three independent repeats of triplicated experiments.

Confocal Laser Scanning Microscopy (CLSM)

m-Slide 8 well high ibiTreat chamber slide suitable for confocal microscopy (ibiTreat: #1.5 polymer coverslip, tissue culture-treated, sterilized) were seeded overnight with A549 (3000 cells in 200 mL RPMI). Cells were then treated with MnCNDs (200 µg mL-1) for 24 h (at 37 °C, 5 % CO2, 95 % air), followed by washing with PBS (4x). For simultaneous colocalization, the MnCDs-pretreated cells were also incubated with 1x solution of CellMask™ Deep Red (Invitrogen), a cell membrane marker, for 10 minutes. Cells were again washed with PBS (4x) prior to fixing them with 4% formaldehyde solution (buffered, pH 6.9) for 8 minutes, which was again followed by washing with PBS (4x).

Images were collected with a light scanning microscope (Zeiss 900) using 20x objective. Since MnCDs are blue emissive (λ_{ex} 405/BP 400-555 nm), all the scanning parameters (laser power,

pinhole size, master gain) employed for treated cells were first established on controls (untreated cells), settled where blue autofluorescence was not detectable. Meanwhile, the signal given by the CellMask™ Deep Red was simultaneously imaged by using the 640 nm laser (detection wavelength: 555-700 nm). The same scanning parameters were then employed to image treated cells. All images were analyzed and processed with Zen Lite 2.0.

Flow cytometry

For the concentration-dependent cell uptake experiments, A549 cells (250000 cells in 1 mL RPMI) were seeded (24 h) on a 6-well plate before treating them with different concentration of MnCDs (500, 100, 200 μg·mL^{-1}) for 24 h (at 37°C, 5% CO_2, 95% air). The cells were then washed with PBS (3x) before treating them with 100 μL 1x Trypsin for 5 minutes. After cell detachment, 1 mL of RPMI was added on the cells, followed by centrifugation (150000 rpm, 5 minutes). The supernatant was discarded and replaced with fresh RPMI to resuspend the cells. The cells were then kept on ice prior to reading with MACSQuant® Analyzer 10 (Miltenyi Biotec). The single-cell population was first gated in a plot of FSC-Height against FSC-Area, prior to obtaining a histogram from the VioGreen Channel (λ_{ex}=405 nm/ BP 525/50 nm) which could detect the fluorescence of the MnCDs. Data analysis was performed by MACSQuantify™ (Version 2.13. 3+164.g7acae8a3) and FCSalyzer.

Cellular uptake mechanism

To assess the energy-dependent internalization of pMnCNDs, two treatment groups were prepared to compare the internalization under physiological conditions (37°C) and at lower temperatures (4°C). A549 cells (10000 cells in 100 μL RPMI) were first seeded on a 96-well plate and incubated for 24 h at 37°C, 5% CO_2, 95% air. For the control group, the medium was replaced with 200 μg·mL^{-1} pMnCNDs solution in RPMI followed by additional 2 hour-incubation at 37°C. The second group was first pre-incubated at 4°C for 30 minutes before replacing the medium with the pMnCNDs solution (200 μg·mL^{-1} in RPMI) and further incubated at 4°C for 2 more hours. The cells were then washed with PBS (3x) and detached with 75 μL 1x Trypsin for 5 minutes. RPMI was added, followed by centrifugation at 300 RCF for 5 minutes. The supernatant was discarded, and the cell pellet was resuspended in 150 μL of medium, in round bottom 96-well plates, and ready for flow cytometry analysis. Herein, the mean fluorescence intensities (MFI) were recorded, and the cell uptake efficiency was calculated as follows:[2]

$$Cell\ uptake\ efficiency\ (\%) = \left(\frac{MFI_{sample} - MFI_{blank}}{MFI_{positive\ control} - MFI_{blank}}\right) \times 100$$

The mechanism of cellular uptake was also studied with the same technique. A549 cells (10000 cells in 100 μL RPMI) were seeded on a 96-well plate and incubated for 24 h at 37°C, 5% CO_2, 95% air. The cells were then treated with endocytosis inhibitors: amiloride (50, 100, 250 μM),

methyl-β-cyclodextrin (50, 100, 250 μM) and chloroquine (50, 100, 250 μM), and cytochalasin D (5, 10, 15 μM) for 30 minutes. The medium containing the inhibitors were then replaced with pMnCND solution in inhibitor-containing RPMI (200 $\mu g \cdot mL^{-1}$) for further co-incubation for 2 h (at 37°C, 5% CO_2, 95% air). The cells were then harvested and analyzed by flow cytometry, as performed previously. The efficiency of cell uptake was also measured with the equation above.

In vivo studies

In vivo MR imaging was carried out on C57BL/6JRj male mice were eight weeks old (20 mg body weight) from Janvier, France. Anesthesia was induced with 4% isoflurane in 30% oxygen and maintained with 1-2% isoflurane during the imaging period. Temperature control (anal) and respiration (through a respiratory pad) were continuously registered along the experiment. Experiments were performed on a 7 T Bruker Biospec 70/30 USR MRI system (Bruker Biospin GmbH, Ettlingen, Germany), interfaced to an AVANCE III console. A BGA12 imaging gradient (maximum gradient strength 400 mT/m) system with a 40 mm diameter quadrature volume resonator was used for MRI data acquisition. MRI was performed using a volumetric array for full mouse body consisting in a volume transmit coil + 4-channel volumetric phased array for reception from Neos Biotec, Pamplona, Spain.

METHOD = <Bruker:RAREVTR> PROTOCOL ['<T1map_RARE>']
TE effective = 7 ms, RARE factor= 2
FOV = 80 x 40 mm, ACQ Matrix = 320 x 160, RECO Matrix = 320 x 160
Slice Thickness = 1.00 mm, N Slices= 14
Number of TR= 8
TR times= [6500.0, 3000.0, 1500.0, 1000.0, 800.0, 600.0, 450.0, 310.0]
Averages= 2
Repetitions= 1
ACQ Time= <0h28m19s200ms>
METHOD = <Bruker:MSME> PROTOCOL ['<T2map_MSME>']
TR/TE = 5500 / 9 ms, FOV = 80 x 40 mm, ACQ Matrix = 320 x 160, RECO Matrix = 320 x 160, Slice Thickness = 1.00 mm, N Slices= 16
N Echoes= 30
T Echoes= [9.0, 18.0, 27.0, 36.0, 45.0, 54.0, 63.0, 72.0, 81.0, 90.0, 99.0, 108.0, 117.0, 126.0, 135.0, 144.0, 153.0, 162.0, 171.0, 180.0, 189.0, 198.0, 207.0, 216.0, 225.0, 234.0, 243.0, 252.0, 261.0, 270.0]
Averages= 1
Repetitions= 1
ACQ Time= <0h10m54s500ms>

At the end of the experiments, organs were harvested for ex vivo analysis. Immediately main organs (heart, liver, spleen, kidneys lungs and brain) were fixed with formalin solution, neutral

buffer, 10%. After 24 hours, the buffer was changed with ethanol 70% (v/v). All animal studies were performed at CIC biomaGUNE. Animals were maintained and handled following the Guidelines for Accommodation and Care of Animals (European Convention for the Protection of Vertebrate Animals Used for Experimental and Other Scientific Purposes). All animal procedures were performed under the European Union Animal Directive (2010/63/EU). Experimental procedures were approved by the local Ethical Committee of CIC biomaGUNE and by the local authorities (Diputación Foral de Guipúzcoa, project number PRO-AE-SS-225).

Long-term toxicity studies

Nine male C57BL/6JRj mice (8 weeks old) were used for the in vivo toxicity study. Animals were housed under standard laboratory conditions with ad libitum access to food and water. All experimental procedures were approved by the local Ethical Committee of CIC biomaGUNE and by the local authorities (Diputación Foral de Guipúzcoa, project number PRO-AE-SS-225).
MnCNDs were administered to all animals. Biological samples were collected at four time points: day 0 (baseline, prior to administration); day 6 post-administration; day 18 post-administration and day 28 post-administration.
Blood samples were obtained at each time point via saphenous vein puncture. Blood was allowed to clot at room temperature and subsequently centrifuged to obtain serum.
Urine samples were collected manually by gentle abdominal massage. All samples were analyzed at CIC bioGUNE (Bilbao, Spain). The following serum parameters were quantified: i) creatinine, ii) urea, iii) GPT (ALT), iv) GOT (AST) and amylase. In urine, total protein levels were measured. All analyses were performed following the standardized protocols of the facility.

Data and Statistical Analysis

When needed, data analyses were performed using either GraphPad Prism 9 or Origin. All data obtained from replicated experiments are reported as mean ± standard deviation (SD), where SD is indicated by error bars. All cellular experiments are performed in duplicate or triplicate and each of these is repeated at least twice independently. n=3 was employed for animal studies. When appliable inhibition values (IC_{50}) were calculated upon fitting the data with a sigmoidal 4PL curve, using GraphPad. When relevant for the discussion, statistical analysis was performed by two-way ANOVA (GraphPad Prism 9) using the following legend for P values: ns when $P > 0.05$, $^{*}P \leq 0.05$, $^{**}P \leq 0.01$, $^{***}P \leq 0.001$, $^{****}P \leq 0.0001$. Long-term toxicity studies were analyzed with GraphPad Prism. Depending on data distribution, either a one-way ANOVA (for normally distributed datasets) or a Kruskal–Wallis test (for non-parametric datasets) was applied. Statistical significance was reported using NEJM-style p-value formatting.

Supporting Figures

Figure S1: A) Microwave temperature (red), pressure (blue) and power (green) profile of cMnCNDs synthesis. B) ^{1}H NMR of cMnCNDs compared to reagents Glutaraldehyde, β-Alanine and Na_2EDTA, C) HPLC-SEC fluorescence (λ_{ex}=350nm, λ_{em}=420 nm, black) and absorbance (λ_{abs}=220 nm, red) chromatograms of cMnCNDs. D) HPLC-SEC bands collection of cMnCNDs. E) Gel Electrophoresis analysis of crude (c) and pure (p) MnCNDs (cMnCND and pMnCND). TAE 1x, 120 V, 20 min. F) Macroscopic difference between c and pMnCND after lyophilization: both materials are orange-brown powders with only minor differences in shade observed. G): Zeta Potential of pMnCNDs solution in FBS buffer at t=0 hours (black line) and t=24 hours (red line). The value observed at t_0=-5.5 mV is essentially unchanged after 24 hours, corresponding to t_{24h}=-5.1 mV.

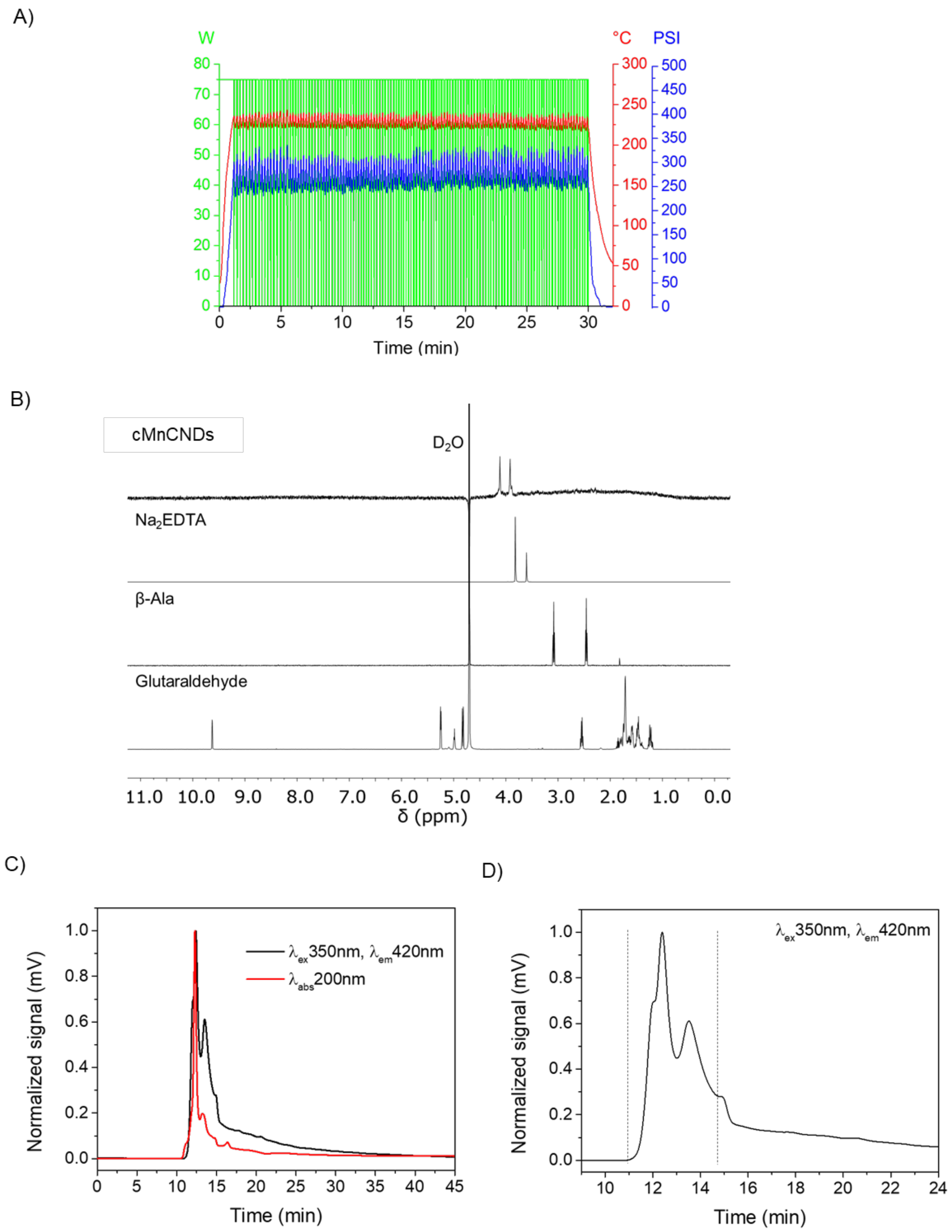

E)

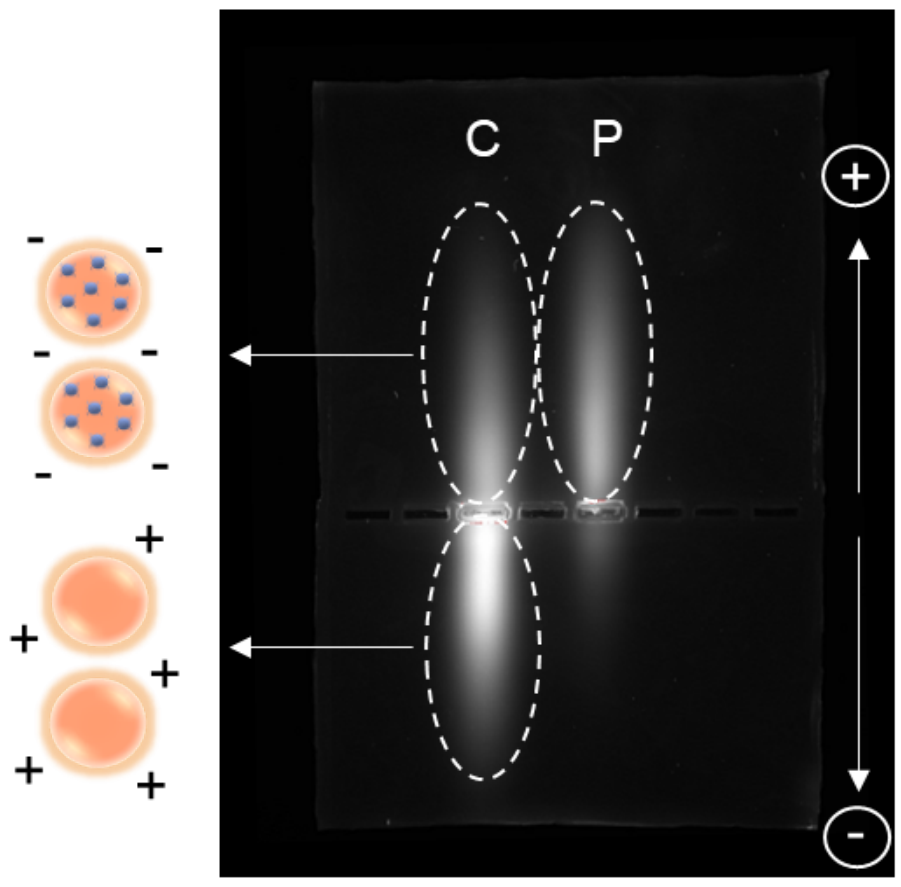

F)

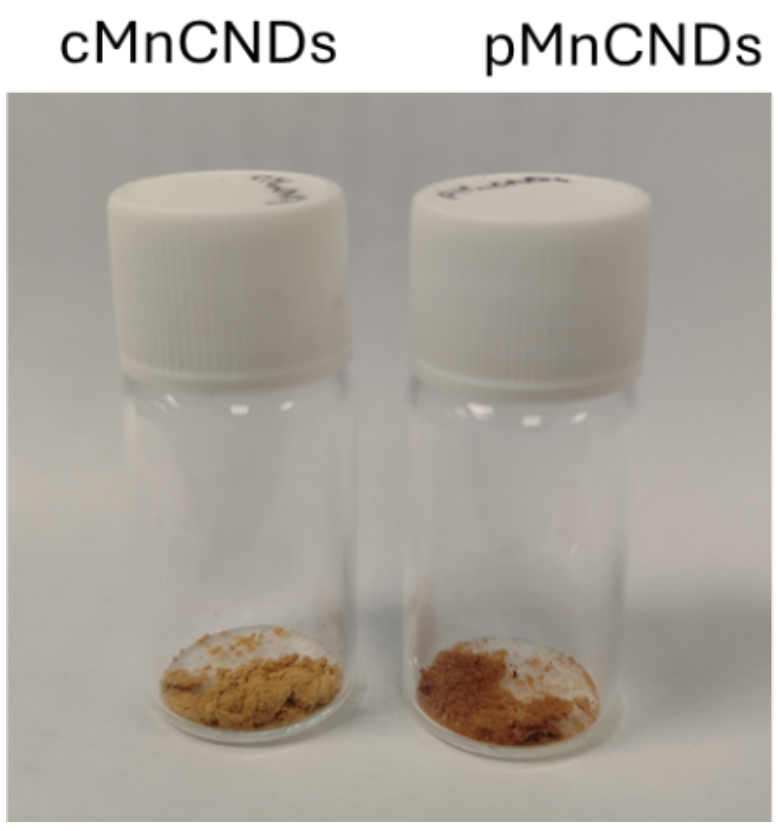

G)

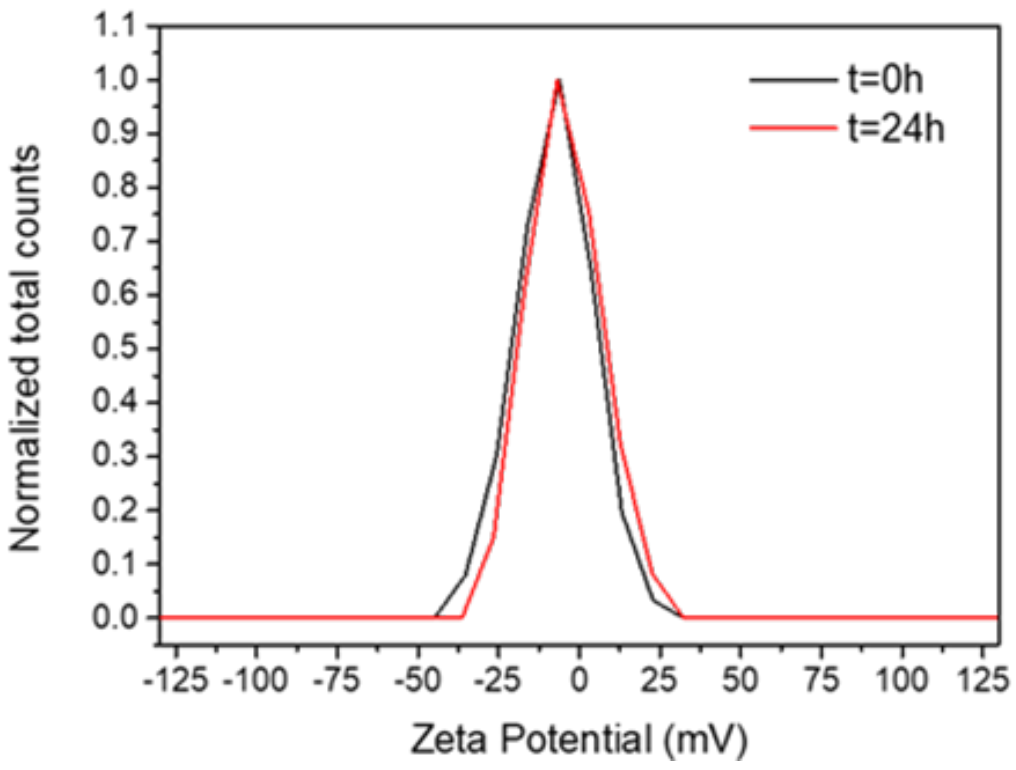

Figure S2: A) Absorption (black), excitation (red) and emission (blue) with λ_{ex}=400nm of cMnCNDs. B) Fluorescence maps of cMnCNDs.

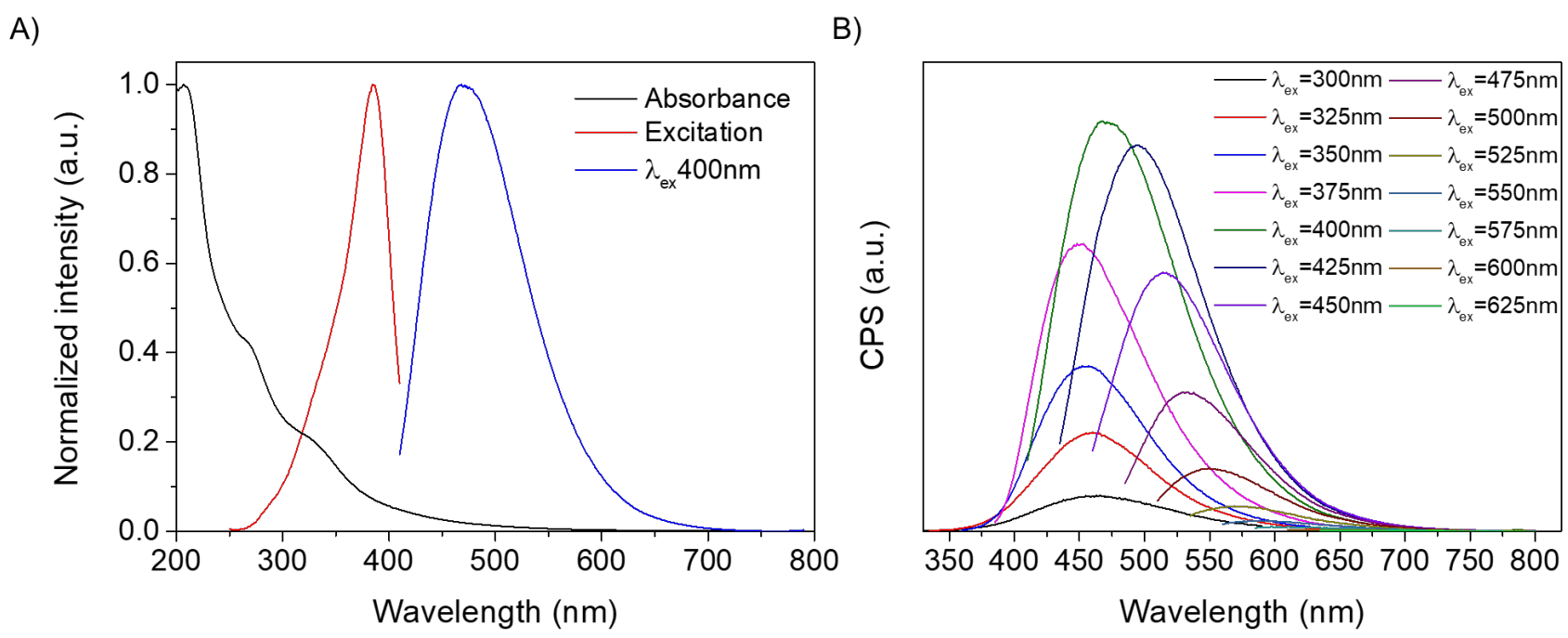

Figure S3: A) AFM image and B) statistical distribution of cMnCNDs.

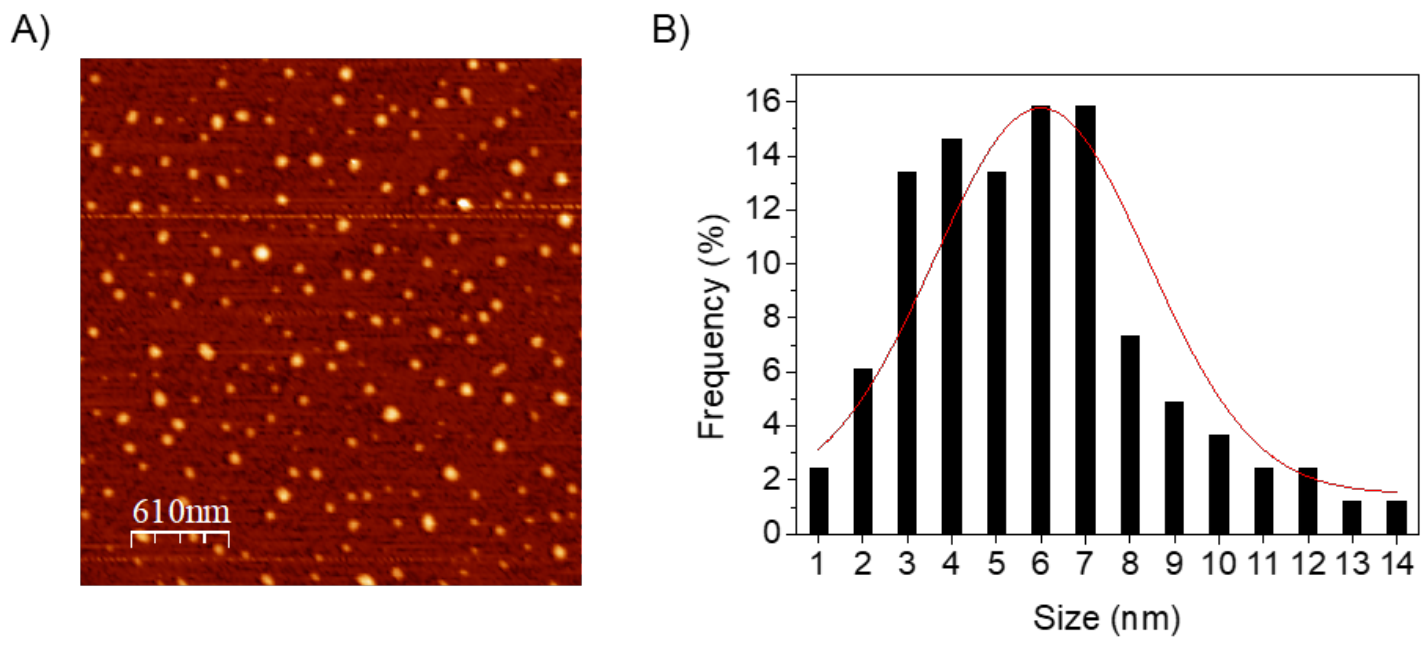

Figure S4: Electron Microscopy analysis

A_ Additional TEM images of pMnCNDs and HR-TEM.

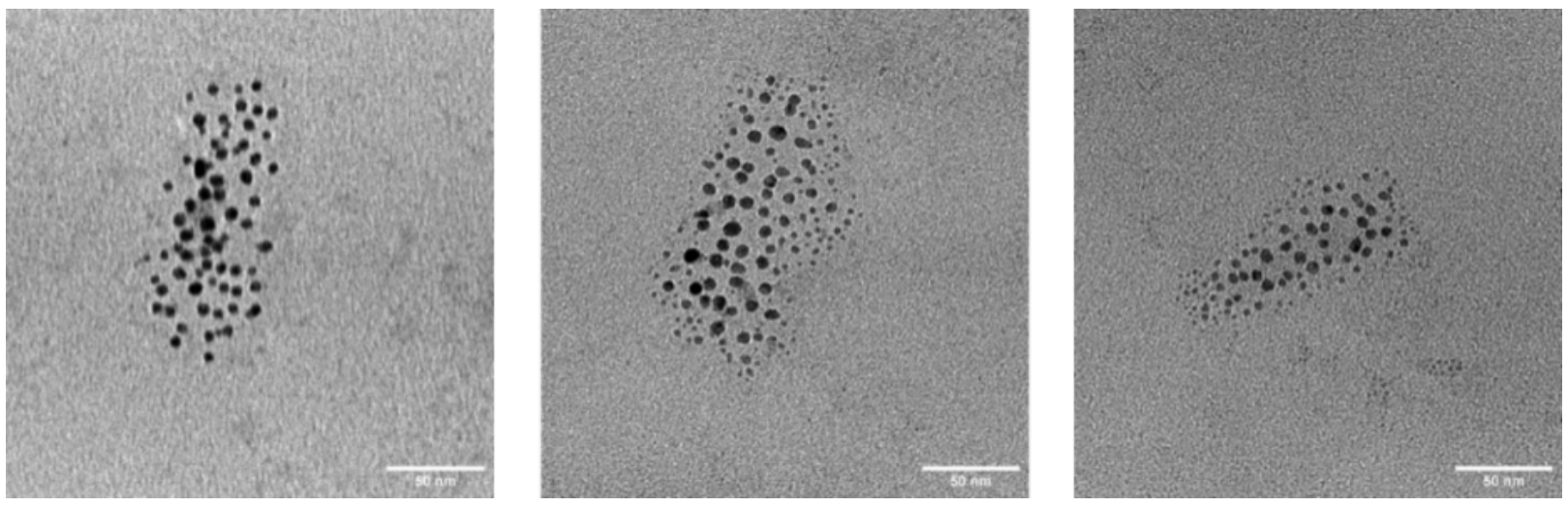

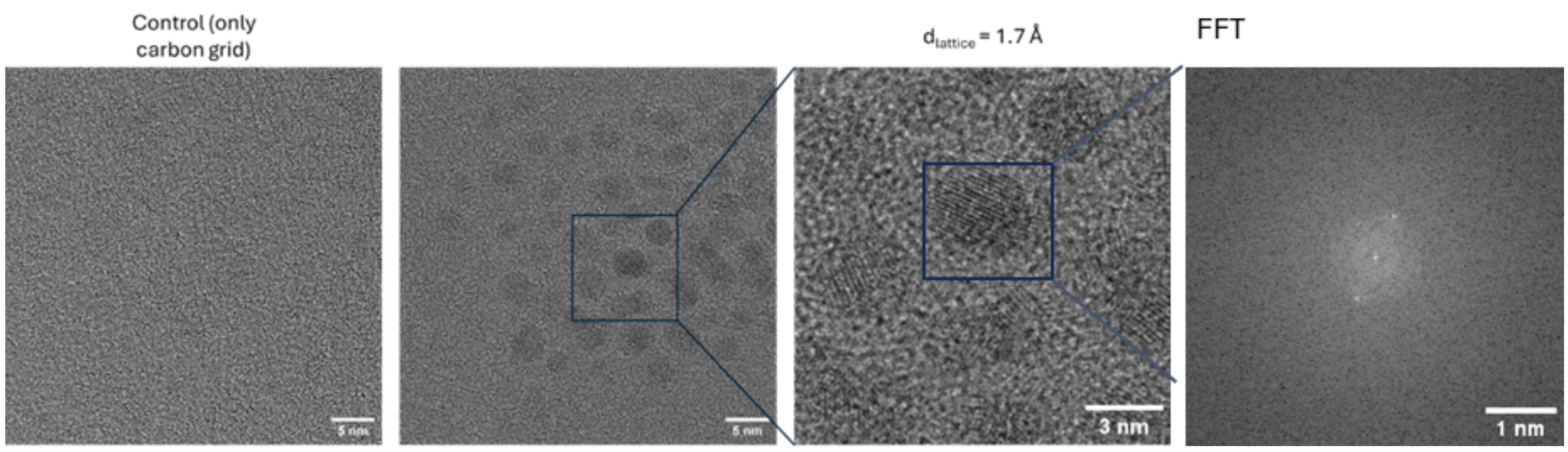

The images below show the changes occurring upon prolonged beam exposure. Although these observations are qualitative, they suggest that the few graphitic features detected during HR-TEM may be at least partially induced by beam irradiation rather than representing the intrinsic structure of the material.

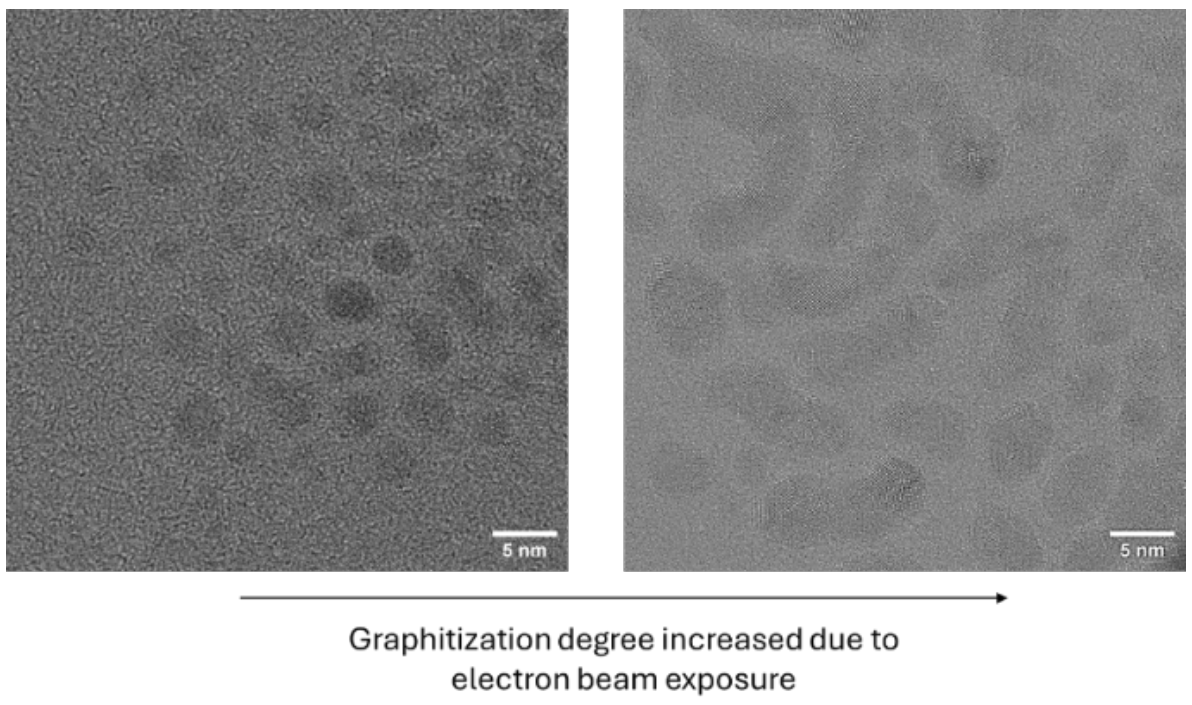

B_ Additional STEM images of pMnCNDs at different magnifications.

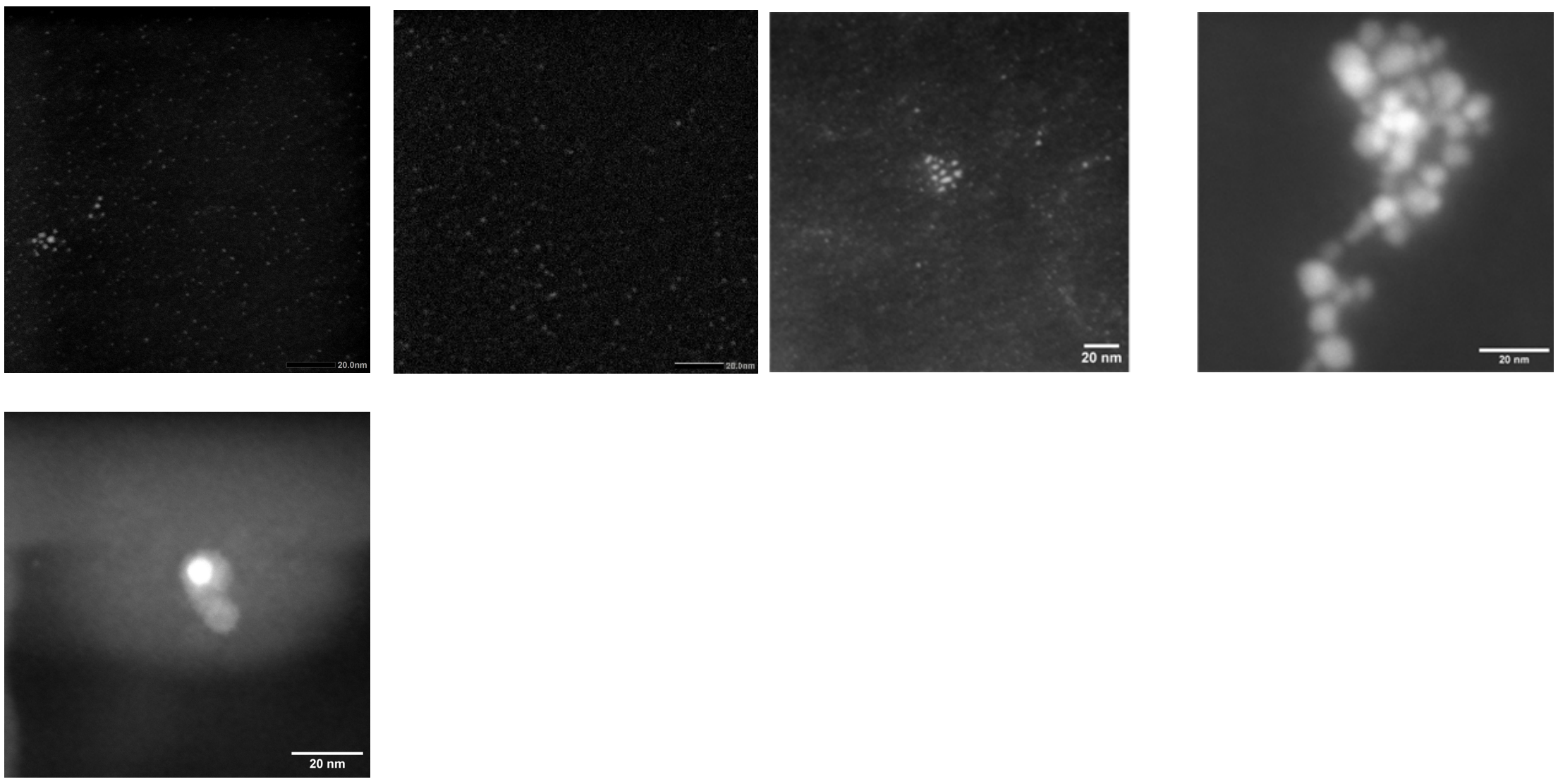

C_Line-scan STEM-EDX analysis.

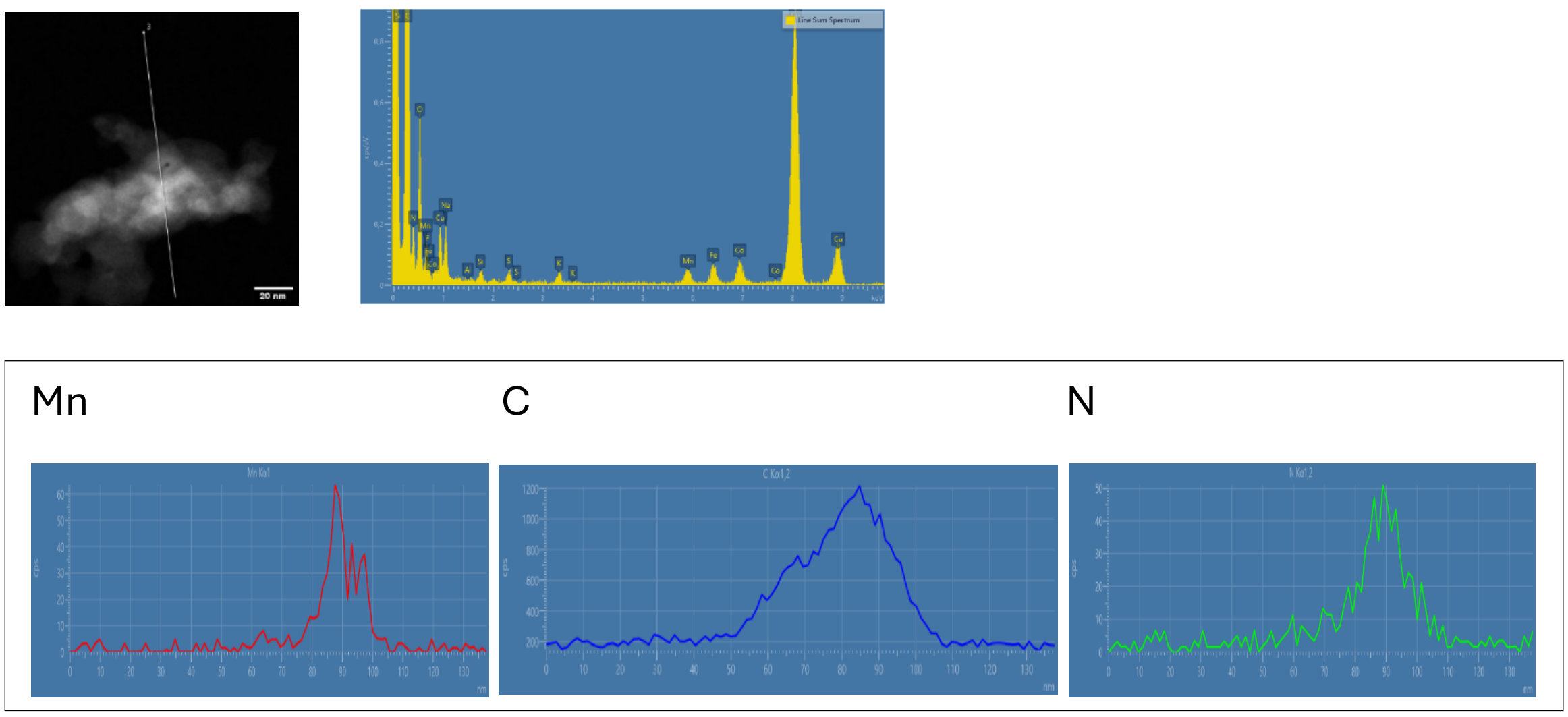

Above on the left: selected line employed for the scan, across particle cluster. Above on the right: energy-dispersive X-ray spectroscopy (EDX) spectrum showing X-ray intensity (counts) as a function of X-ray energy (keV). Below: EDX line profile showing the spatial distribution of Mn, C, and N (Kα) intensities along the selected line across the particle cluster. As the analysis was performed on carbon-film-supported copper grids, the carbon profile exhibits a relatively high background signal originating from the support film.

Nevertheless, co-localization of Mn, C, and N along the scanned region is evident. The carbon signal extends over a slightly broader region (approximately 50–110 nm), whereas the Mn signal is more spatially confined (approximately 75–110 nm), indicating that Mn is distributed within a subset of the carbon-based framework rather than across the entire scanned area.

Below other representative examples:

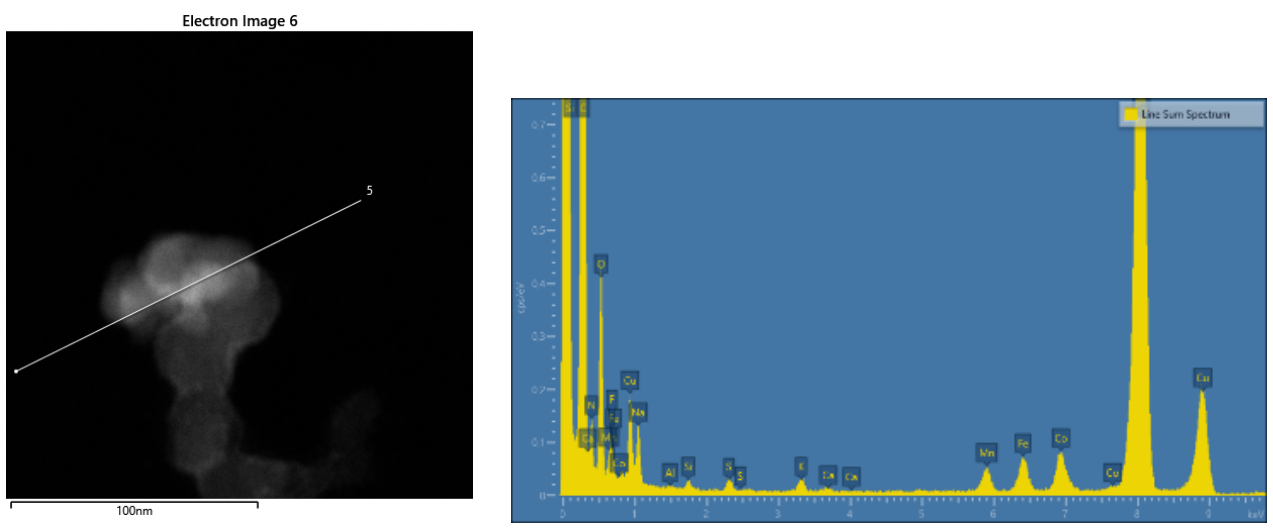

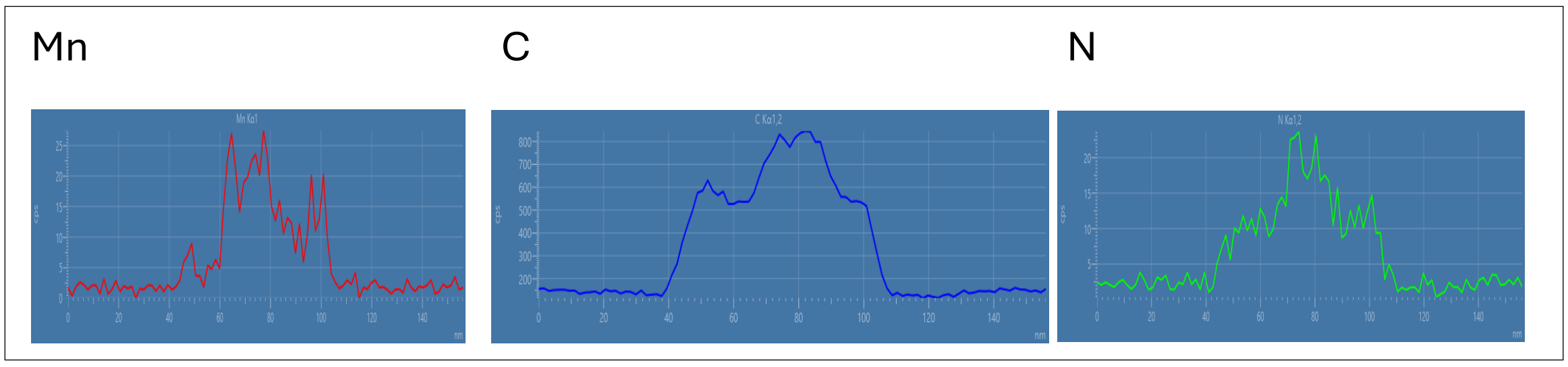

Figure S5: A)¹H NMR of cMnCNDs and pMnCNDs. 500 MHz, D_2O, water suppression sequence. B) DOSY NMR of pMnCNDs. 500 MHz, D_2O.

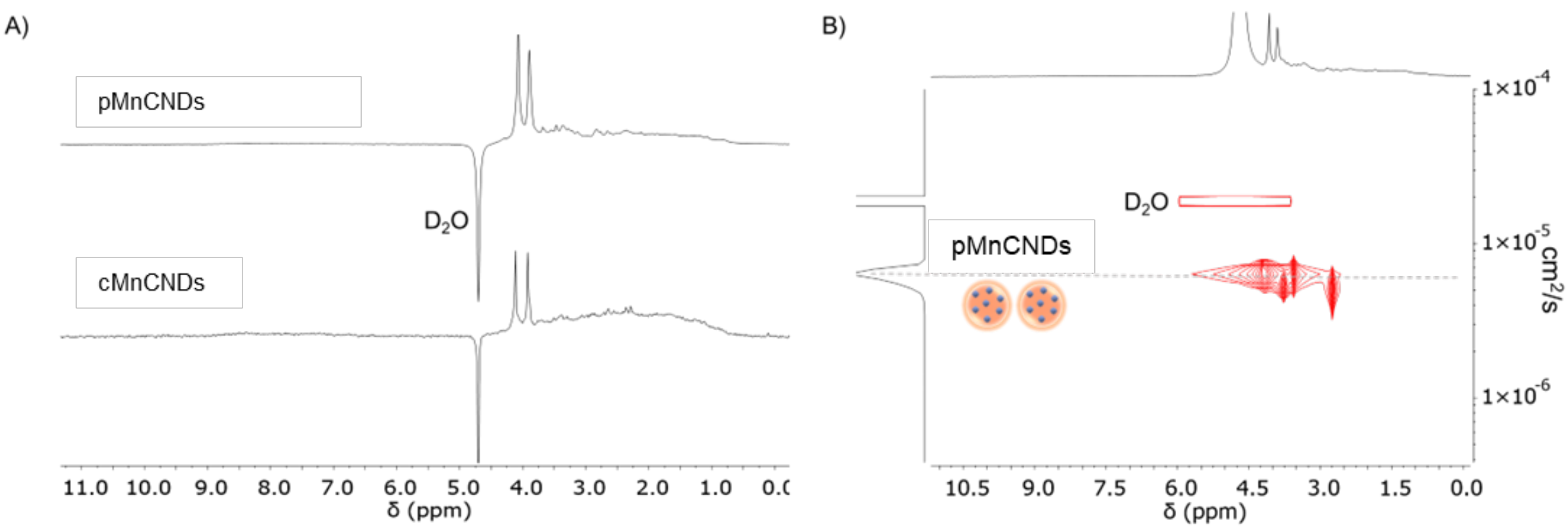

Figure S6: Deconvolution and curve-fitting of A) C_{1s}, B) O_{1s} and C) N_{1s} signals of pMnCND by XPS. cMnCND was analyzed by XPS for comparison: D) survey spectrum; deconvolution and curve fitting of E) C_{1s}, F) Mn_{3s} and G) Mn_{2p}.

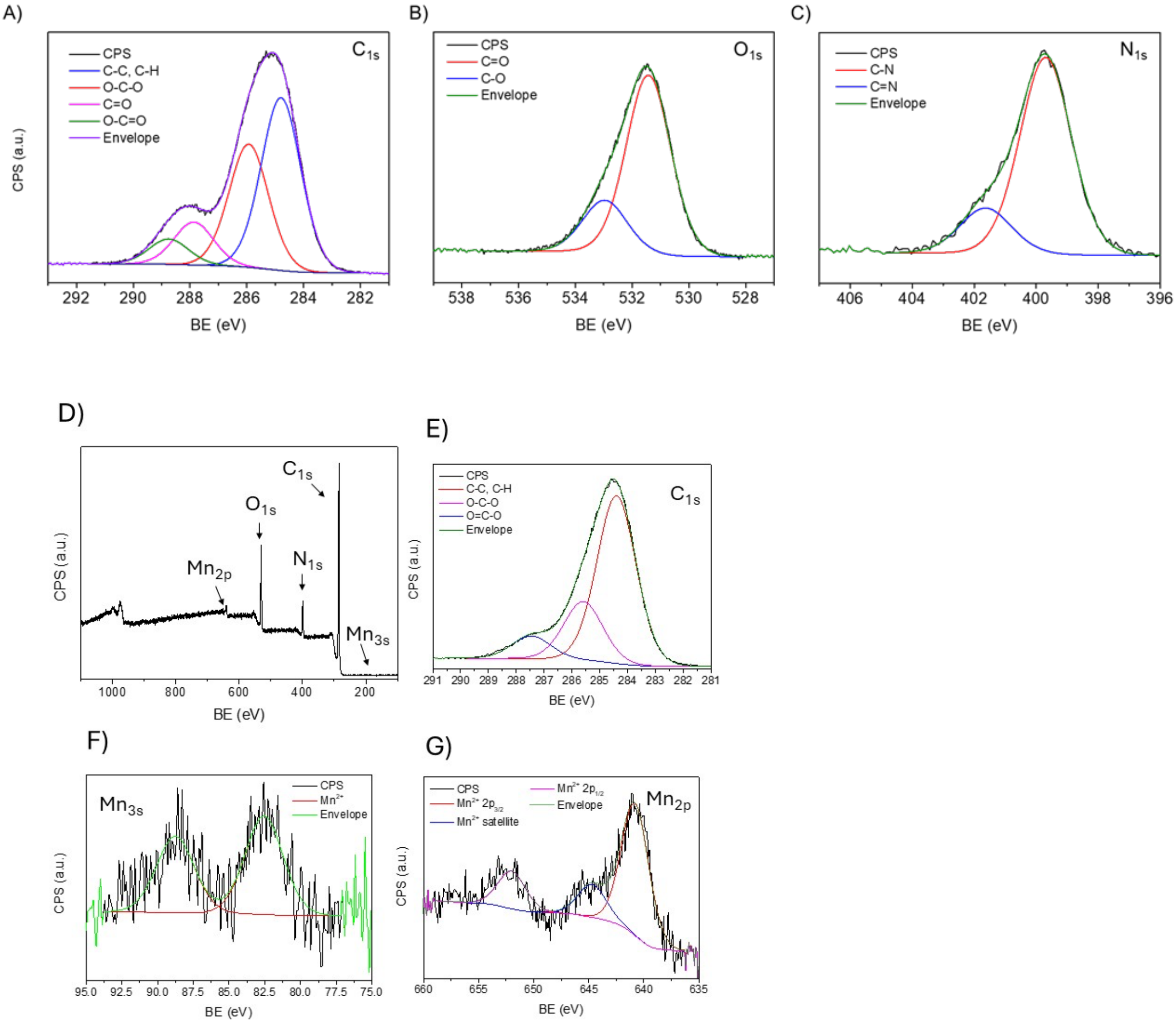

Figure S7: A) FT-IR spectra of cMnCNDs and pMnCNDs. Both spectra show a broad band at 3675-2340 cm^{-1} resulting from the stretching vibration of C-H, N-H and O-H bonds. Additionally, pMnCNDs exhibits a minor signal at approximately 2930 cm^{-1}, probably related to aliphatic stretching of CH_2 and CH_3 groups. The band around 1570 cm^{-1} is ascribed to the stretching vibration of C=O of O=C-O^- groups. It indicates that both the materials possess deprotonated carboxylic acid, probably involved in metal chelation. A weak signal is observed at 1440 cm^{-1} which can be produced by aliphatic vibration of CH_2 and CH_3 groups. The absorption band at about 1390 cm^{-1} arises from the stretching modes of C-O, C-C and C-N of the amorphous organic framework. The band at around 1230 cm^{-1} belongs to the stretching vibration of C-N, while the peak at 1055 cm^{-1} is related to C-O stretching vibrations. B) pH titration curve of pMnCNDs. [pMnCNDs] = 1 mg·mL^{-1}.

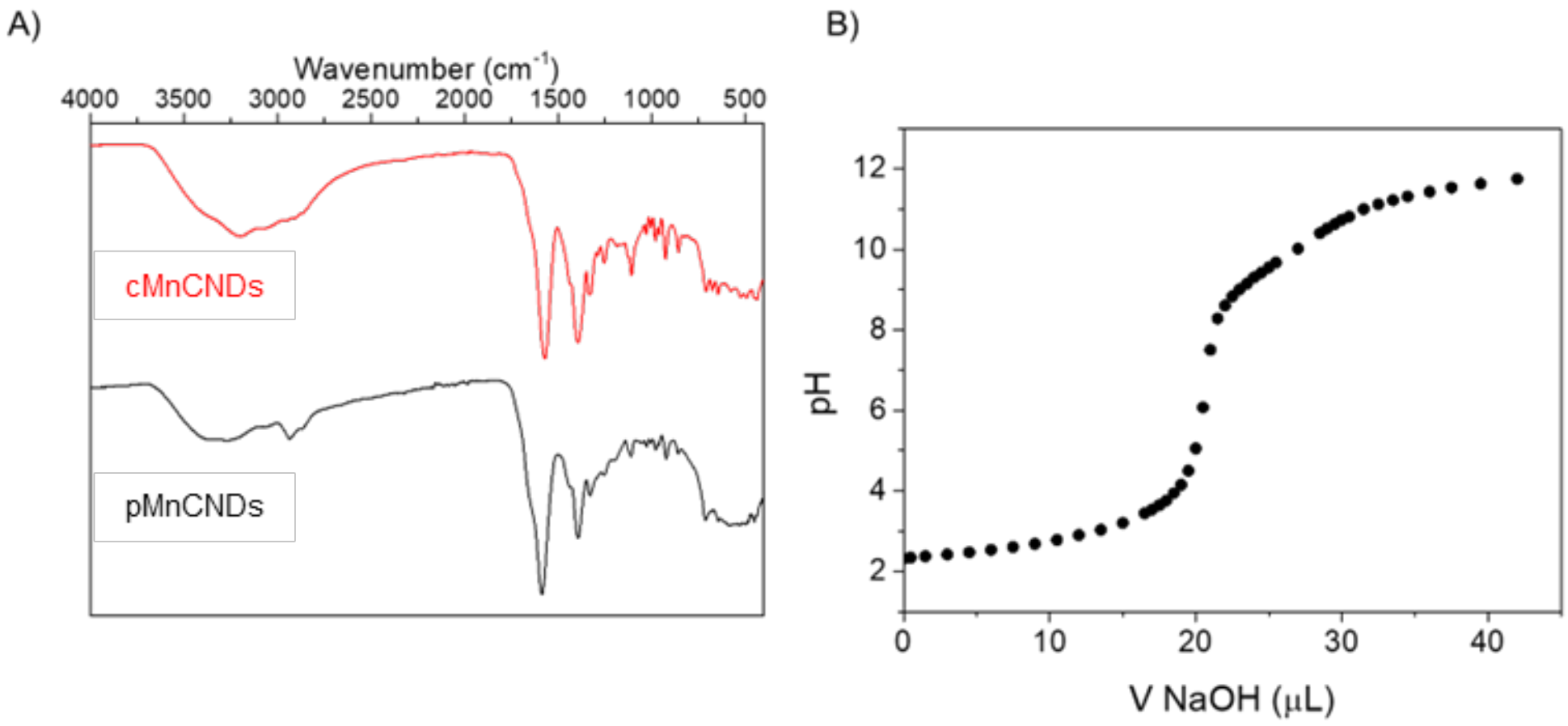

Figure S8: A) Relaxivity r_2 of pMnCNDs at 1.5 and 7T. B) Evolution of the relative water proton paramagnetic transversal relaxation rate $R_2p(t)/R_2p(0)$ vs. time for pMnCNDs. 1.5 T. C) Stability of pMnCNDs at pH 2.8, 4.2, 6.2, 7.4 and 9.7 in $NH_4^+HCOO^-$ buffer after 24h. The values remained unchanged for 1 week (1.5 T).

D), E), F) and G). Time-dependent change of the paramagnetic contribution to the longitudinal and transverse relaxation rates of water protons, normalized to their initial value (R1p(t)/R1p(0) and R2p(t)/R2p(0) vs. time (days)) for pMnCNDs in cellular medium (D) and E), RPMI complemented with 10%FBS) and plasma (F) and G)).

H), I), J), K) relaxivities r_1 and r_2 of cMnCNDs (water, 1.5 T) at 0 and 72h, showing stability as well as longitudinal relaxivity 2 folds lower compared to pMnCNDs

L), M) relaxivities r_1 and r_2 of $MnCl_2{\cdot}4H_2O$ (water, 1.5 T)

N) relaxivities r_1 and r_2 of Gadovist (Gadobutrol supplied by Merck, water, 1.5 T)

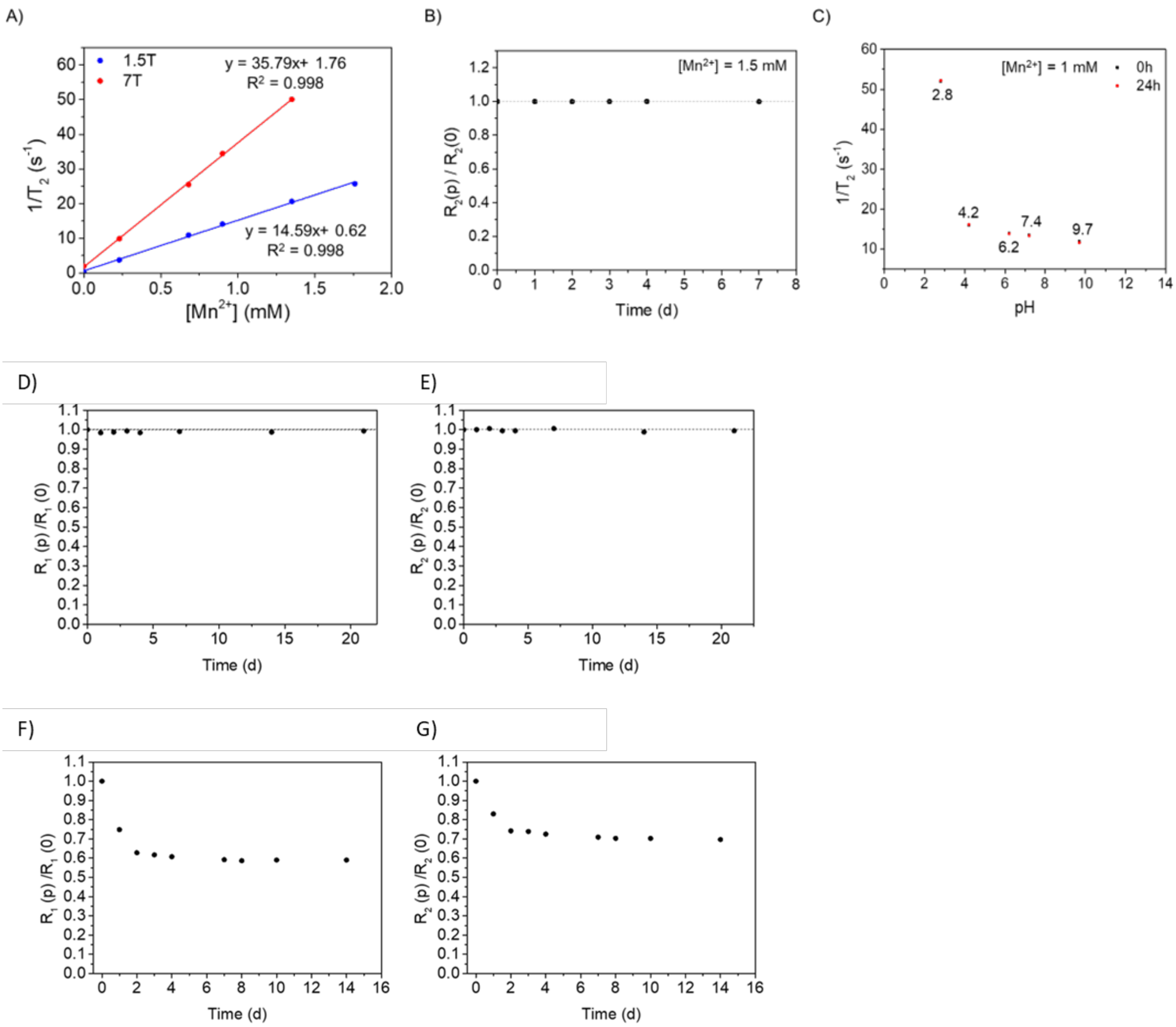

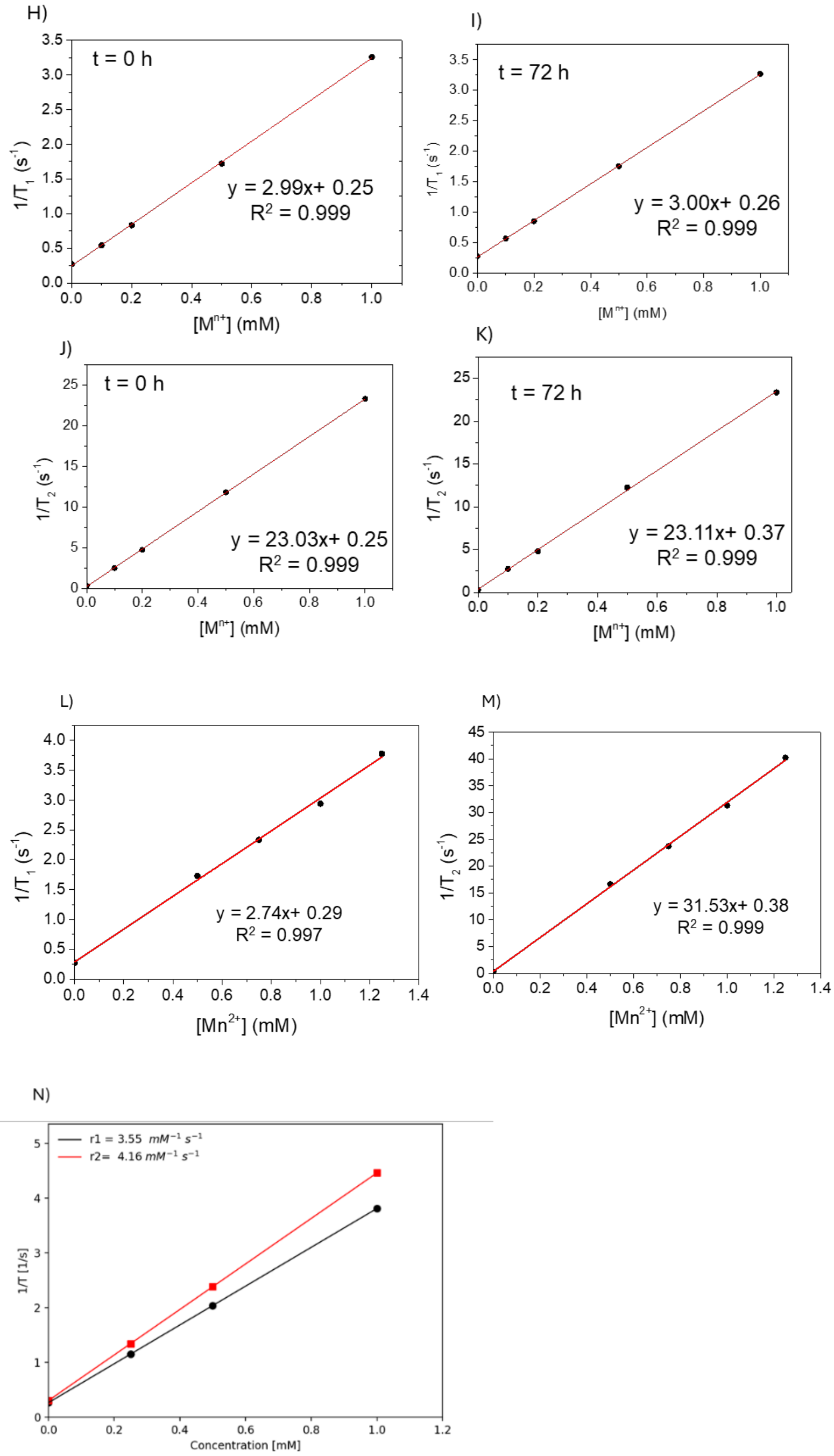
H)
t = 0 h
y = 2.99x+ 0.25
R2 = 0.999
1/T1 (s-1)
[Mn+] (mM)
I)
t = 72 h
y = 3.00x+ 0.26
R2 = 0.999
1/T1 (s-1)
[Mn+] (mM)
J)
t = 0 h
y = 23.03x+ 0.25
R2 = 0.999
1/T2 (s-1)
[Mn+] (mM)
K)
t = 72 h
y = 23.11x+ 0.37
R2 = 0.999
1/T2 (s-1)
[Mn+] (mM)
L)
y = 2.74x+ 0.29
R2 = 0.997
1/T1 (s-1)
[Mn2+] (mM)
M)
y = 31.53x+ 0.38
R2 = 0.999
1/T2 (s-1)
[Mn2+] (mM)
N)
r1 = 3.55 mM-1 s-1
r2= 4.16 mM-1 s-1
1/T [1/s]
Concentration [mM]

Figure S9: Additional cellular studies.

S9_A

Cell viability (by MTT assay) of HDFa (human dermal fibroblast) cell lines treated with pMnCNDs and cMnCNDs, for 24 h and 72h. Cell viability remained above 80% in all the tested conditions.

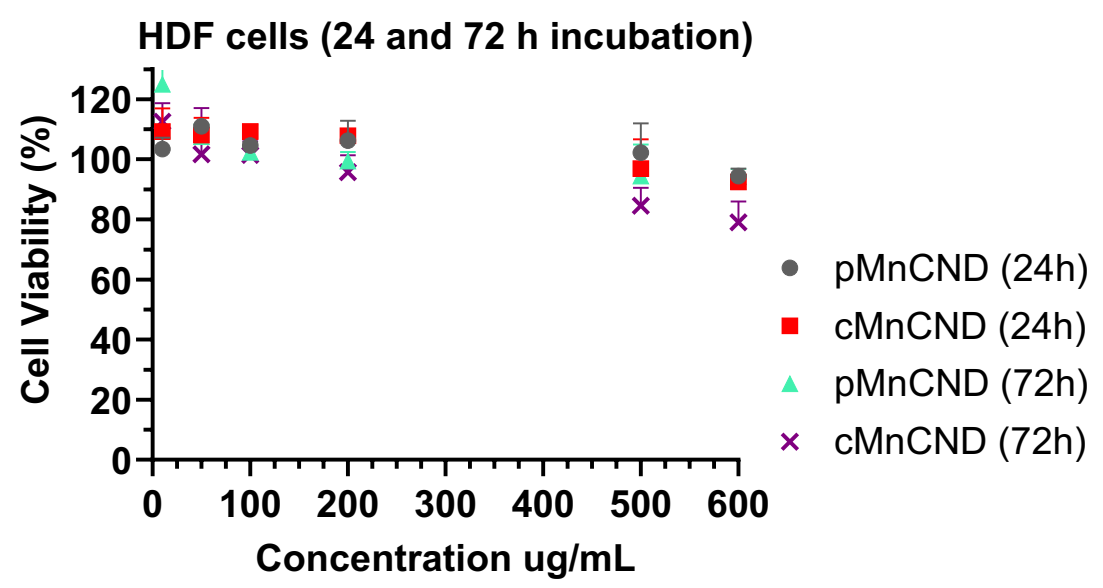

S9_B

The IC_{50} of $MnCl_2$ in A549 cells has been reported to be approximately 100 $\mu g \cdot mL^{-1}$. In our *in vitro* experiments, cells are typically treated with 200 $\mu g \cdot mL^{-1}$ pMnCNDs, corresponding to approximately 10 $\mu g \cdot mL^{-1}$ Mn (≈10-fold lower than the reported IC_{50}).

In this control experiment, A549 cells were treated for 24 h with either 200 $\mu g \cdot mL^{-1}$ pMnCNDs, 10 $\mu g \cdot mL^{-1}$ $MnCl_2$(-A), or 100 $\mu g \cdot mL^{-1}$ $MnCl_2$ (-B), followed by washing and analysis of the cellular pellet by ICP-MS. As expected, Mn uptake was markedly higher at 100 $\mu g \cdot mL^{-1}$ $MnCl_2$ compared with all other conditions. However, when comparing metal uptake delivered by pMnCNDs with the equivalent concentration of free $MnCl_2$ (10 $\mu g \cdot mL^{-1}$), the nanomaterial enabled greater intracellular Mn accumulation.

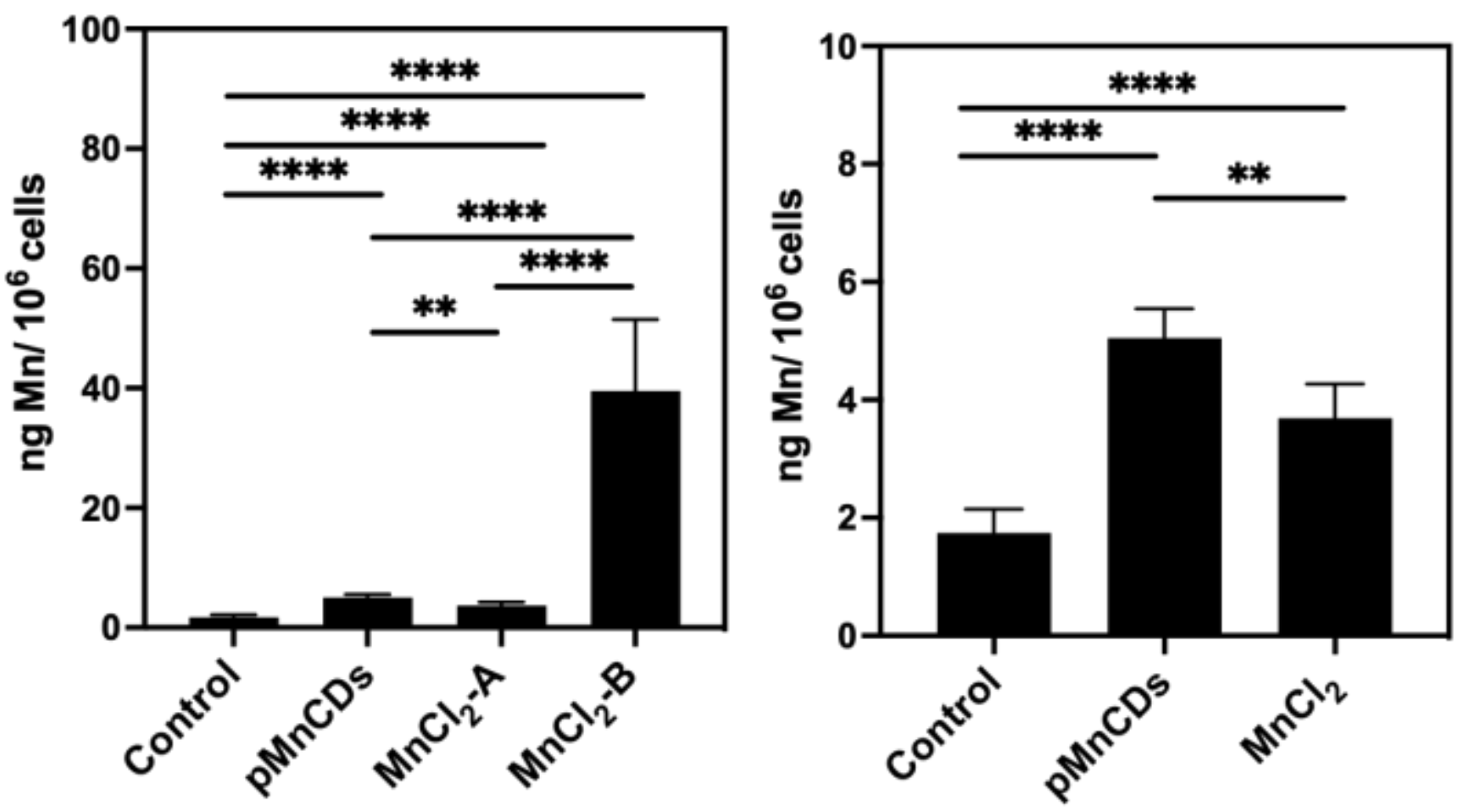

S9_C

Confocal images of live A) Ctrl on top left and B) A549 cells treated with 200 μg·mL^{-1} of pMnCNDs for 24 h (λ_{ex} = 405 nm, λ_{em} = 420-480 nm); from left to right: bright field (BF), blue emission, merged images of blue emission+BF. The scale bar is 100 μm. B) Mean grey value of Ctrl and treated cells.

A)

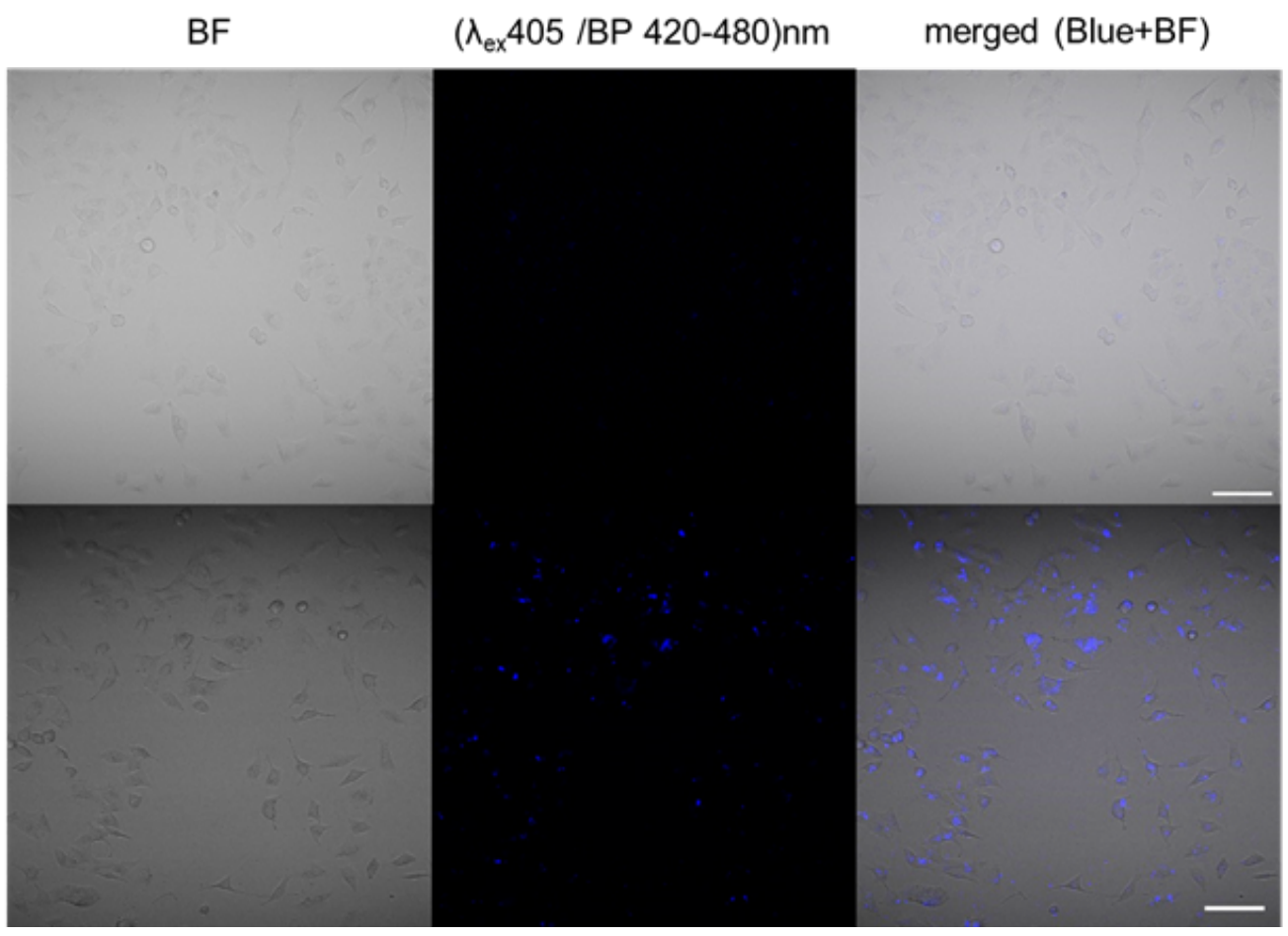

B)

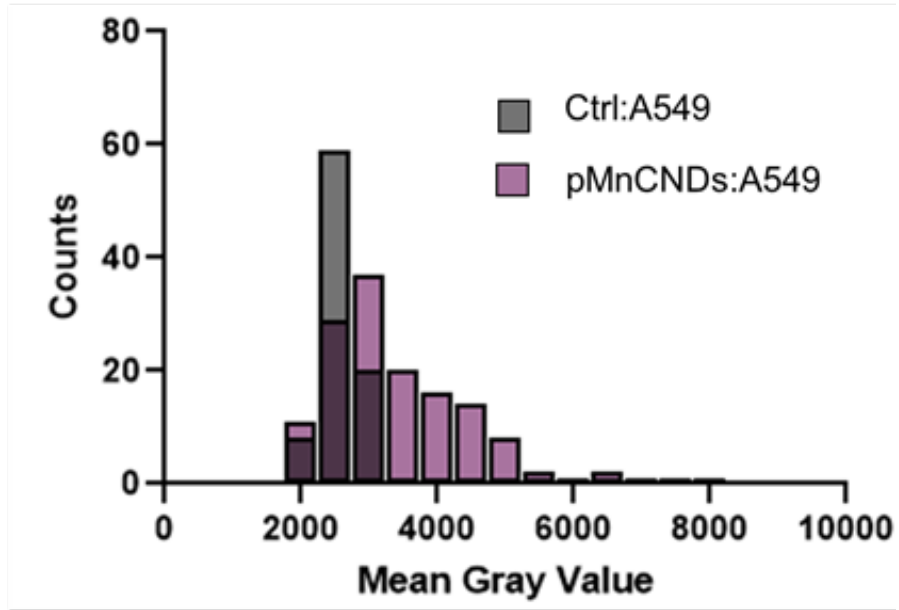

Figure S10: Additional CLSM images (20x air) of fixed A549 control cells. From left to right: bright field (BF), blue emission (λ_{ex}=405 nm/ BP 400-555 nm), red emission (λ_{ex}=405 nm/ BP 555-700 nm), and merged images of blue+red+BF of non-treated A549 cells stained with CellMask DeepRed.

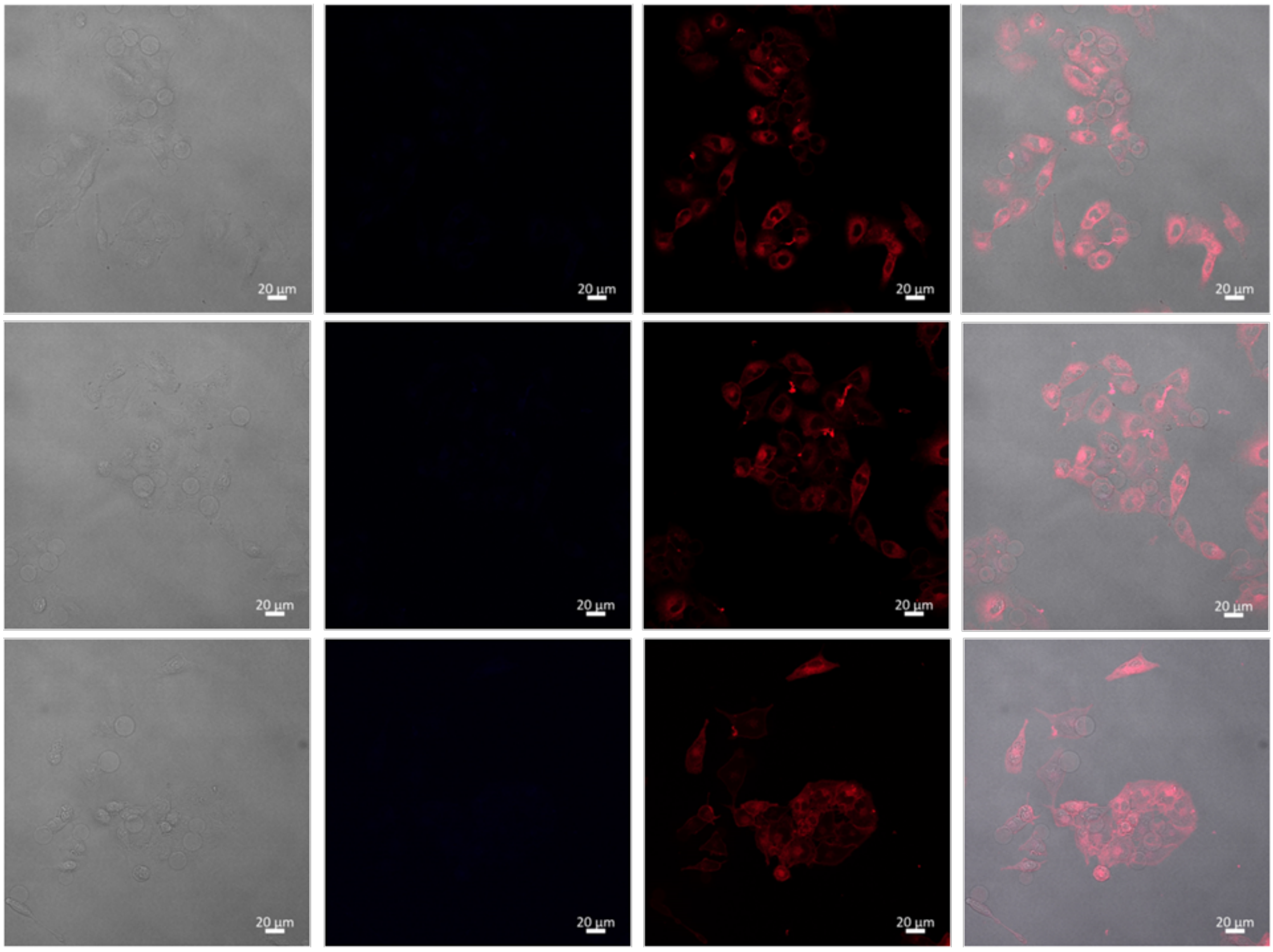

Figure S11: Additional CLSM images (20x air) of fixed A549 cells treated with 200 µg mL-1 of pMnCNDs for 24h. From left to right: bright field (BF), blue emission (λex=405 nm/ BP 400-555 nm), red emission (λex=405 nm/ BP 555-700 nm), and merged images of blue+red+BF of treated A549 cells stained with CellMask DeepRed.

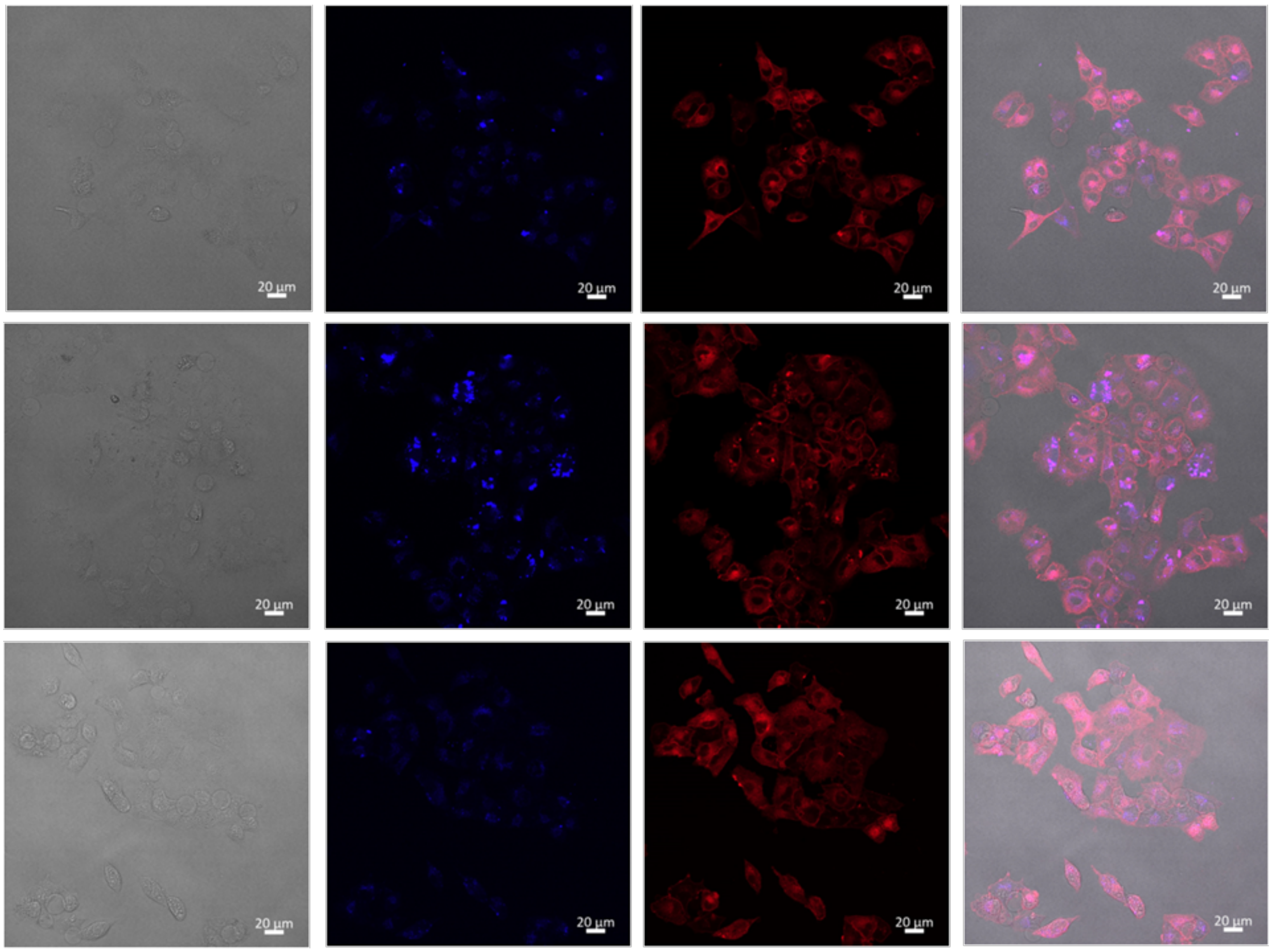

Figure S12: MTT results on A549 cells by different cell uptake inhibitors of different concentrations (50 to 1000 µM): cytochalasin D, amiloride, methyl-β-cyclodextrin, and chloroquine. The values are expressed as mean ± SD (n=15).

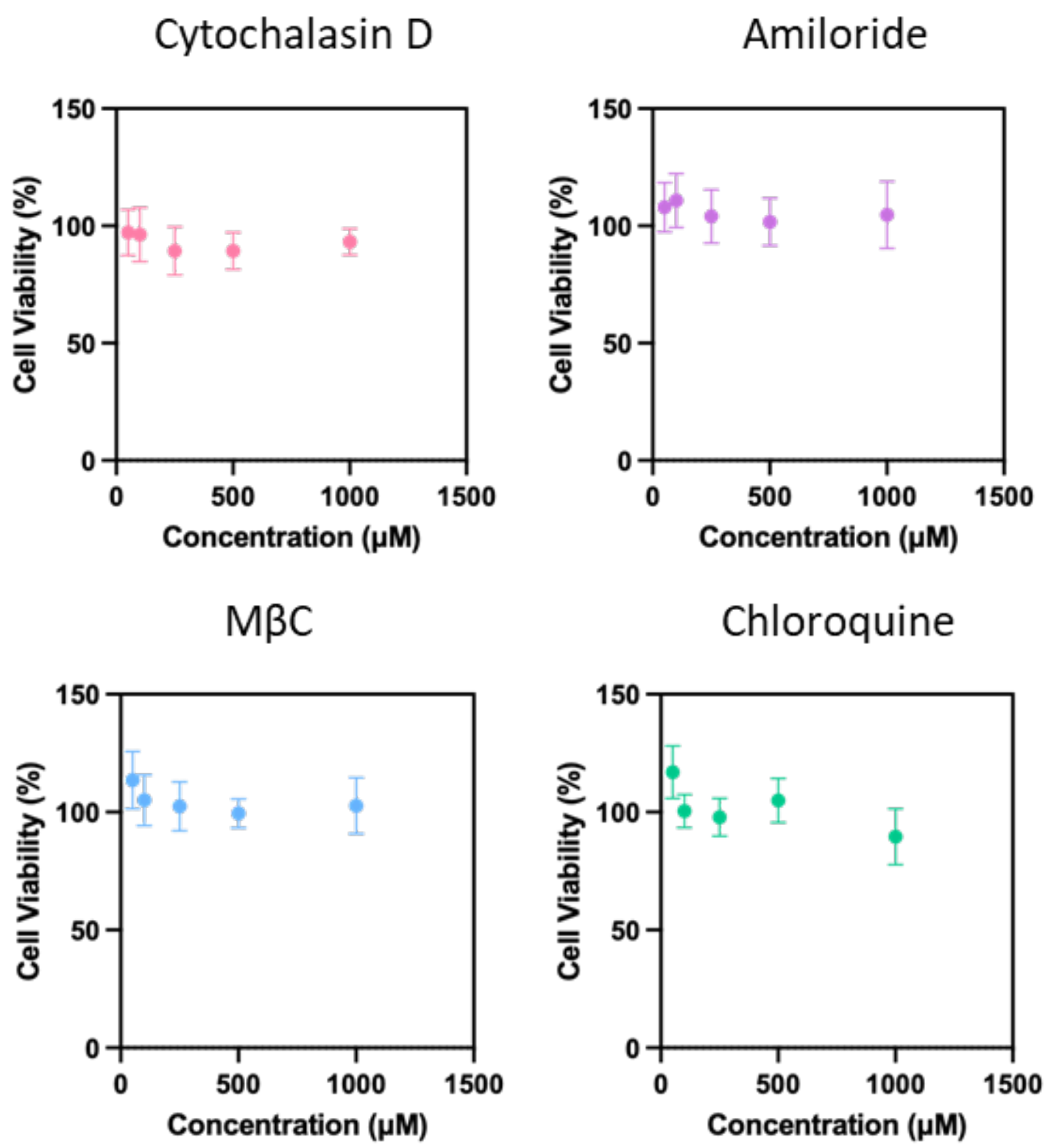

Figure S13: T_2 relaxation values of the analyzed organs before and after administration of pMnCNDs (n=3, results are the mean±SD).

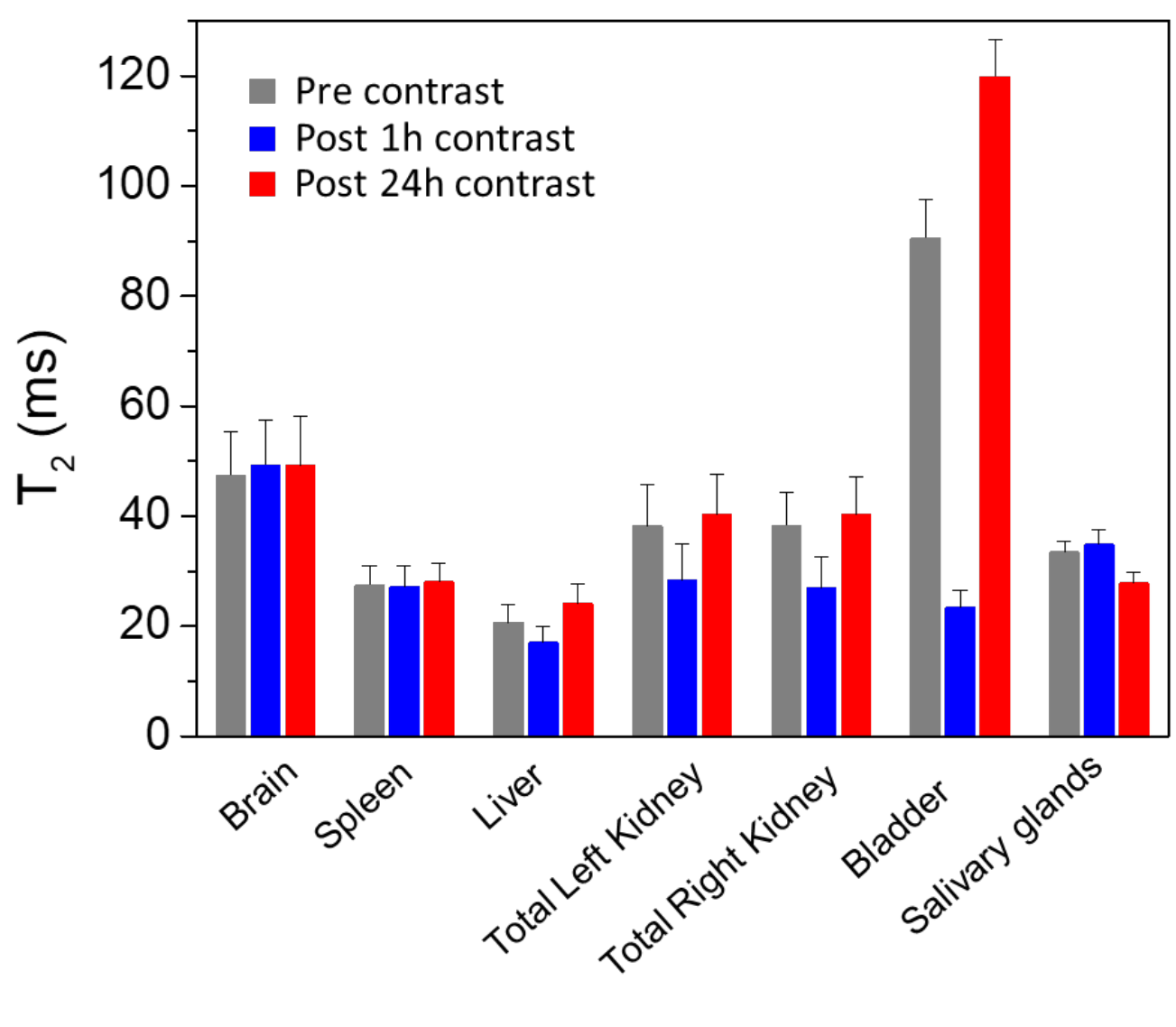

Figure S14: Collected urine before, 1h and 24h post administration of pMnCNDs (left); percentage of manganese found in urines before and after 90min, 24h, 48h, 72h and 6d.

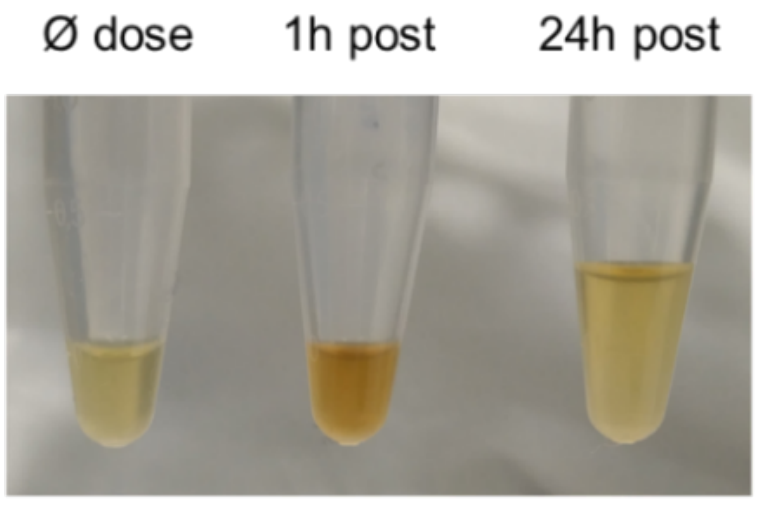

Urine	[Mn] %
Control	-
90 minutes	22
24 hours	-
48 hours	-
72 hours	-
6 days	-

Figure S15: Percentage of manganese found in faeces before and after, 24h, 48h and 6d.

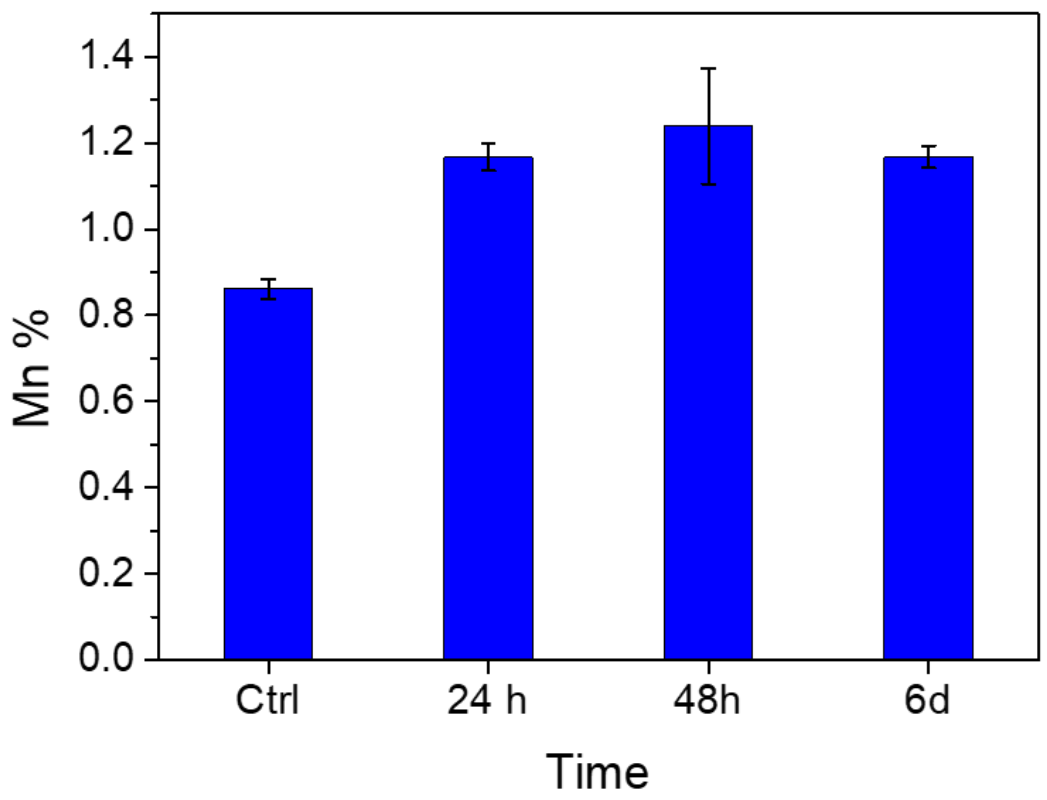

Figure S16: T_1 relaxation maps (water-green color) MRI images of salivary glands after injection of pMnCNDs at time points 24h, 48h, 72h and 6d (0.075 mmol Mn·Kg^{-1}).

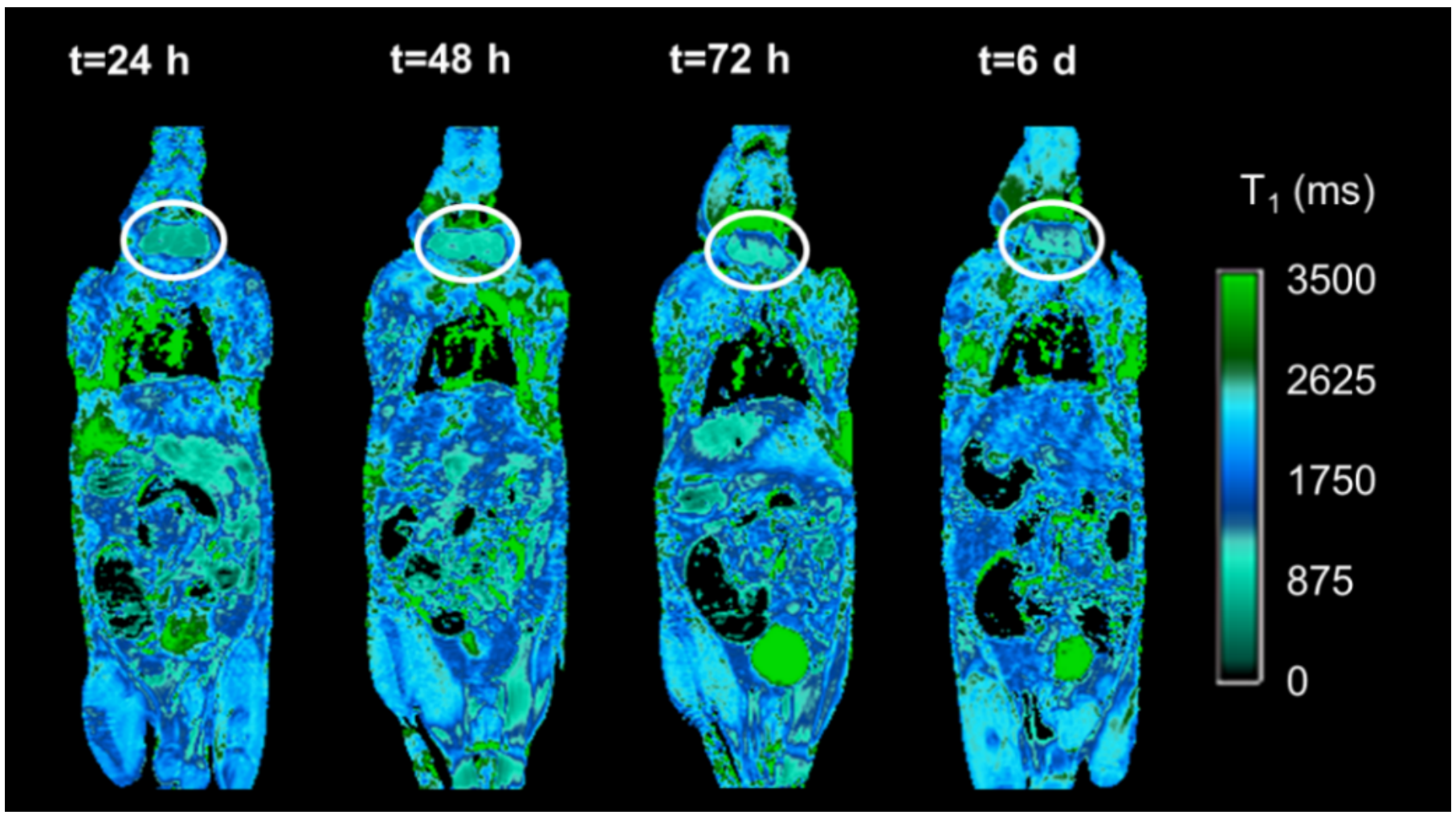

Bibliography

(1) Cardo, L.; Martínez-Parra, L.; Cesco, M.; Echeverría-Beistegui, B. M.; Martínez-Moro, M.; Herrero-Álvarez, N.; Cabrerizo, M. B.; Carregal-Romero, S.; Ramos-Cabrer, P.; Ruiz-Cabello, J.; et al. *Small* **2023**, 19, 2206442

(2) Liu, L.; Chemistry, B. P.; Biology, C. *Peptide Macrocycles*; **2022**, 301–316
